# Identification of catabolite control protein A from *Staphylococcus aureus* as a target of silver ions†
†Electronic supplementary information (ESI) available: Experimental procedures, supplementary figures and tables. See DOI: 10.1039/c7sc02251d


**DOI:** 10.1039/c7sc02251d

**Published:** 2017-10-02

**Authors:** Xiangwen Liao, Fang Yang, Runming Wang, Xiaojun He, Hongyan Li, Richard Y. T. Kao, Wei Xia, Hongzhe Sun

**Affiliations:** a MOE Key Laboratory of Bioinorganic and Synthetic Chemistry , School of Chemistry , Sun Yat-sen University , Guangzhou , 510275 , China . Email: xiawei5@mail.sysu.edu.cn; b Hunan Provincial Key Laboratory for Ethnic Dong Medicine Research , Hunan University of Medicine , Huaihua , 418000 , China; c Department of Chemistry , The University of Hong Kong , Pokfulam Road , Hong Kong , P. R. China . Email: hsun@hku.hk; d Department of Microbiology , State Key Laboratory for Emerging Infectious Diseases , The University of Hong Kong , Hong Kong , P. R. China

## Abstract

*Staphylococcus aureus* is one of the most common pathogenic bacteria that causes human infectious diseases. The emergence of antibiotic-resistant strains of *S. aureus* promotes the development of new anti-bacterial strategies. Silver ions (Ag^+^) have attracted profound attention due to their broad-spectrum antimicrobial activities. Although the antibacterial properties of silver have been well known for many centuries, its mechanism of action remains unclear and its protein targets are rarely reported. Herein, we identify the catabolite control protein A (CcpA) of *S. aureus* as a putative target for Ag^+^. CcpA binds 2 molar equivalents of Ag^+^*via* its two cysteine residues (Cys216 and Cys242). Importantly, Ag^+^ binding induces CcpA oligomerization and abolishes its DNA binding capability, which further attenuates *S. aureus* growth and suppresses α-hemolysin toxicity. This study extends our understanding of the bactericidal effects of silver.

## Abstract 2

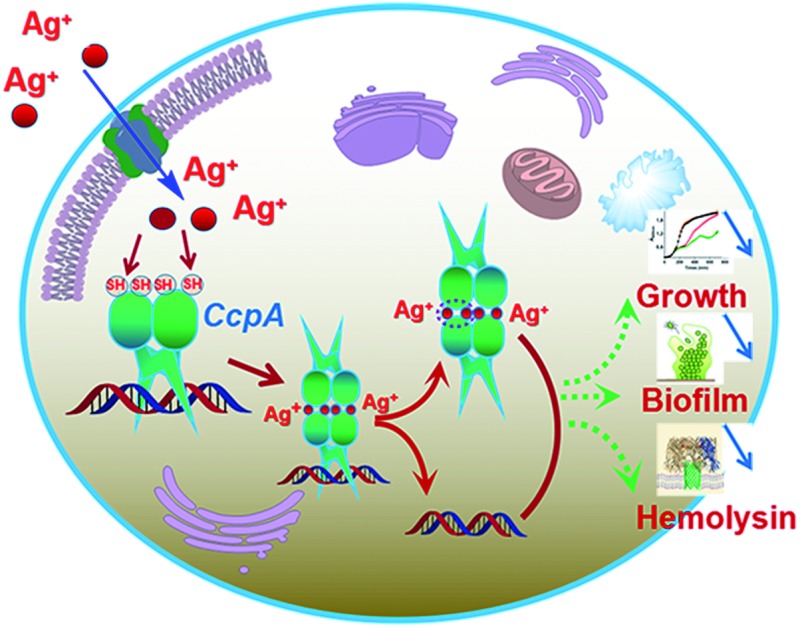
The catabolite control protein A (CcpA) of *S. aureus* has been identified as a putative target for silver ions.

## Introduction

The main carbon catabolite repression (CCR) system is an important global control system of various bacteria, which allows the bacteria to adapt quickly to a preferred carbon source first. This is usually achieved by the repression of genes whose products are involved in the catabolism of alternative, less preferred carbon sources. In Gram-positive bacteria, a highly conserved regulator, catabolite control protein A (CcpA), exerts the important catabolite repression function.^1^ CcpA is usually activated by its co-regulator *via* the formation of a complex which recognizes the catabolite-responsive element (cre) sequences and regulates downstream gene expression.^2^

*Staphylococcus aureus* (*S. aureus*), a worldwide spread human pathogen, is the leading cause of hospital- and community-acquired infections. The pathogen causes a series of human diseases ranging from minor skin infections to life-threatening sepsis.^3^ In particular, the emergence of drug-resistant strains of the bacteria, such as methicillin-resistant and vancomycin-resistant *S. aureus*, poses a huge threat to public health worldwide.^4^,^5^ Intriguingly, *S. aureus* CcpA (*Sa*CcpA) is not only involved in the regulation of carbon metabolism but also affects antibiotic resistance, biofilm formation, toxin expression and even the infectivity of this bacterium, implying its critical role as an important global regulator for bacterial metabolism as well as virulence.^6^–^9^ Recently, small molecule inhibitors targeting the *S. aureus* virulence regulators, SarA or MgrA, are reported to be efficacious in animal models, indicating that targeting these regulator proteins might be a promising anti-bacterial strategy.^10^,^11^ Given the important role that CcpA played in *S. aureus* virulence, this transcription factor could be a feasible anti-bacterial drug target.^12^ Chemical inhibition of CcpA binding to the cre DNA region could potentially diminish *S. aureus* virulence.

Silver ions (Ag^+^) have been used as antibacterial agents for centuries. It is suggested that Ag^+^ could bind to the thiol group (–SH) of bacterial enzymes and subsequently cause enzyme deactivation.^13^ However, up to now, few Ag^+^ protein targets have been identified and characterized. Herein, we demonstrate that *Sa*CcpA serves as a potential target for Ag^+^ in *S. aureus*. Ag^+^ binds specifically to the two cysteines of *Sa*CcpA and abolishes its cre-binding property, which further abrogates *S. aureus* α-hemolysin secretion and biofilm formation.

## Results and discussion

It is reported that silver nanoparticles (AgNPs) could block bacterial sugar metabolism in order to be bactericidal.^14^ Furthermore, recent studies demonstrated that bacterial strains with a TCA cycle genes knockout were less sensitive to Ag^+^ treatment.^15^ All of these data imply that Ag^+^ targets the bacterial central metabolism pathway. CcpA is an important regulator that coordinates central metabolism in Gram-positive bacteria.^1^,^16^ We therefore investigate the possible effect of Ag^+^ on CcpA physiological function.

**Fig. 1:**
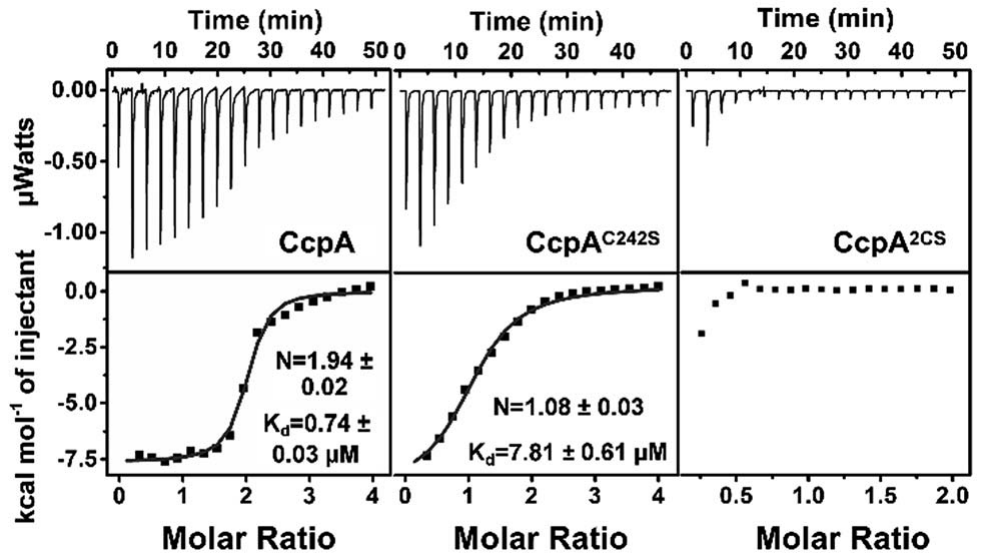
Isothermal titration calorimetry results of Ag^+^ binding to CcpA, CcpA^C242S^ and CcpA^2CS^ in 50 mM Tris-HNO_3_ and 150 mM NaNO_3_ buffer at pH 7.4. The titrations were carried out at 25 °C. The data were fit to a one-set-of-sites binding model using the Origin software.

Sequence alignments of 20 CcpA family proteins from different Gram-positive bacteria species revealed that *Sa*CcpA contains two cysteine residues (Cys216 and Cys242), which are almost absent in other species (Fig. S1†). Given that Ag^+^ is highly thiophilic, we postulated that Ag^+^ could bind to *Sa*CcpA. To test this hypothesis, purified *Sa*CcpA was incubated with 3 molar equivalents of Ag^+^ followed by the removal of excess amounts of Ag^+^ with a desalting column. By using a BCA assay and inductively coupled plasma mass spectrometry (ICP-MS), the stoichiometry of Ag^+^ binding to CcpA (monomer) was determined to be 2 : 1, indicating that each CcpA monomer binds 2 molar equivalents of Ag^+^ (Fig. S2†). Subsequently, we examined whether the two cysteine residues are involved in the Ag^+^ binding. We measured free thiol amounts of the CcpA protein after premixing with different molar ratios of Ag^+^ by Ellman’s assay. As expected, the free thiols of CcpA decreased with increasing pre-mixed Ag^+^ concentrations until the Ag^+^/CcpA molar ratio reached 2 : 1, confirming that the cysteines participate in Ag^+^ binding (Fig. S3†). The two cysteines were then individually mutated to serine. Both the ICP-MS measurement and Ellman’s assay showed that the CcpA^C216S^ and CcpA^C242S^ mutants could bind one Ag^+^ per monomer, while the double Cys mutant CcpA^C2S^ had no Ag^+^ binding capability, indicating that both cysteines are responsible for Ag^+^ binding (Fig. S2 and S3†). In line with the results, isothermal titration calorimetry (ITC) data showed that wild-type (WT) CcpA binds 1.94 ± 0.02 molar equivalents of Ag^+^ with an apparent dissociation constant (*K*_d_) of 0.74 ± 0.03 μM. The single Cys mutant CcpA^C242S^ binds 1.08 ± 0.03 molar equivalents of Ag^+^ with a much lower affinity (*K*_d_ = 7.81 ± 0.61 μM), while the double Cys mutant CcpA^2CS^ had no detectable binding to Ag^+^ (Fig. 1 and Table S2†).

**Fig. 2:**
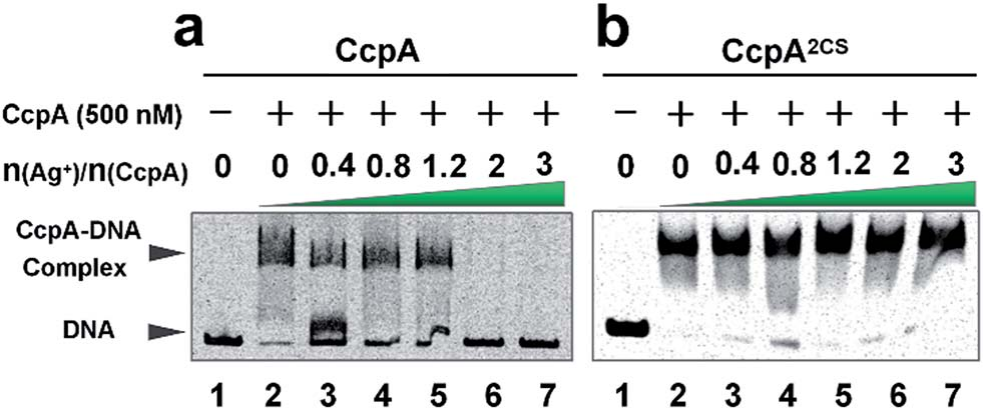
Electrophoretic mobility shift assay (EMSA) of the CcpA binding to the catabolite responsive elements (cre) of the *pckA* gene. Approximately 35 nM *pckA* promoter (189 base pairs) was incubated with 500 nM purified CcpA (a) or CcpA^2CS^ (b) in the presence of 0, 0.4, 0.8, 1.2, 2 and 3 molar equivalents of Ag^+^.

As a global transcription factor, CcpA binds to a couple of gene promoter regions (cre sequence), such as the *pckA* (encoding phosphoenol-pyruvate carboxykinase) and *hla* (encoding α-hemolysin) promoters. To examine the effect of Ag^+^ binding on the CcpA’s function, we investigated whether Ag^+^ affected the CcpA-DNA binding properties *in vitro*. An electrophoretic mobility shift assay (EMSA) was applied to 35 nM *pckA* DNA probe (covers the cre sequence of the *pckA* gene) and a negative control *proC* probe with increasing concentration of CcpA (0–700 nM monomer concentration). As expected, the significant shift of DNA was only observed for *pckA* but not *proC* (Fig. S4a and c†). The results are consistent with a previous report that *Sa*CcpA does not essentially require the association with phosphorylated HPr for efficient DNA binding.^8^ However, the addition of gradient amounts of Ag^+^ obviously disrupted the complex formation (Fig. 2a). The double-mutant CcpA^2CS^ binds *pckA* DNA in a similar way to the WT CcpA (Fig. S4b†). However, the CcpA^2CS^ mutant would not dissociate from the DNA probe even in the presence of Ag^+^ (Fig. 2b). A similar phenomenon was observed for the *hla* probe (Fig. S5†). CcpA binding to the cre region was enhanced by phosphorylated HPr (HPr-P).^8^ However, the EMSA assay demonstrates that Ag^+^ prevented CcpA-DNA binding even in an excess amount of Hpr-P (Fig. S6a†). Native polyacrylamide gel electrophoresis (PAGE) further confirmed that Ag^+^ binding also disrupted the DNA-CcpA-(Hpr-P) ternary complex (Fig. S6b†). All these results demonstrate that Ag^+^ binding completely abolishes CcpA-DNA binding *in vitro*.

**Fig. 3:**
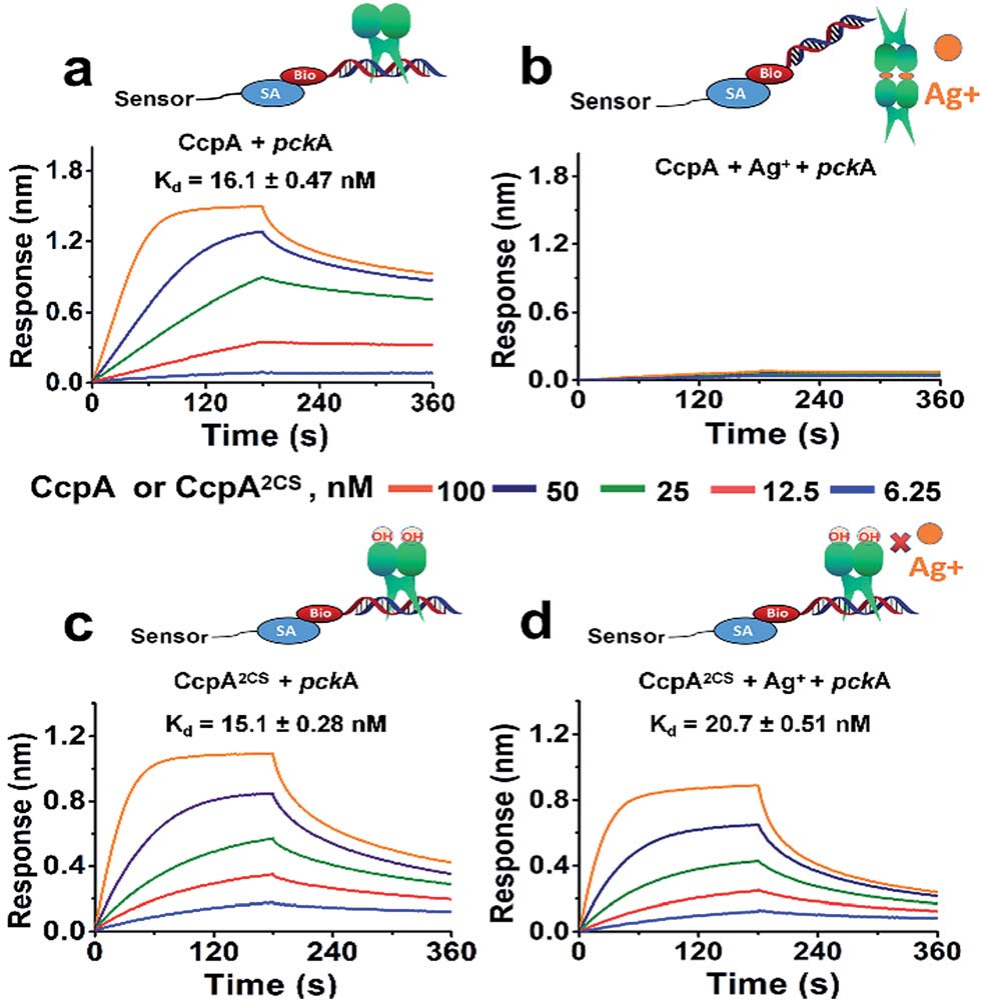
The DNA binding capabilities of CcpA (a), CcpA with Ag^+^ (b), CcpA^2CS^ (c) and CcpA^2CS^ with Ag^+^ (d) were measured by BioLayer Interferometry (BLI). Biotinylated *pckA* (300 nM) was captured on pre-immobilized streptavidin Dip and Read sensor heads for 3 min. Association occurred from 0 to 180 s and dissociation was monitored thereafter for up to 360 s. The *K*_d_ values are presented as the mean ± s.e.m. derived from a global fitting of all binding curves.

For further confirmation, we measured the DNA binding capabilities of the WT CcpA and CcpA^2CS^ mutant by BioLayer Interferometry (BLI). A Biotin-labeled *pckA* DNA probe was immobilized on a streptavidin sensor to enable kinetic analysis of the CcpA binding to the DNA probe. As shown in Fig. 3a, WT CcpA binds strongly to the *pckA* probe with a *K*_d_ value of 16.1 ± 0.47 nM. While in the presence of Ag^+^, the binding of WT CcpA to the *pckA* probe is undetectable (Fig. 3b). In contrast, the binding affinities of CcpA^2CS^ to the *pckA* probe are nearly identical in the absence and presence of Ag^+^, with *K*_d_ values of 15.1 ± 0.28 nM and 20.7 ± 0.51 nM, respectively (Fig. 3c and d and Table S3†). Collectively, these data demonstrate that Ag^+^ binds to the two Cys residues of CcpA, and Ag^+^ binding disrupts its DNA binding capability.

**Fig. 4:**
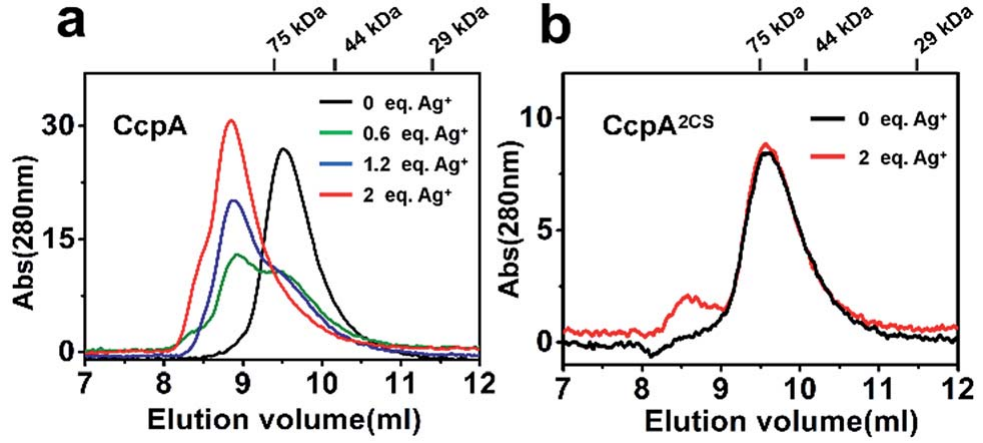
The effects of Ag^+^ binding on the oligomeric states of CcpA (a) and CcpA^2CS^ (b). Size-exclusion chromatography analysis of CcpA incubated with 0, 0.6, 1.2 and 2.0 molar equivalents of Ag^+^ and CcpA^2CS^ incubated with 0 and 2.0 molar equivalents of Ag^+^.

Previous studies demonstrated that the binding of non-physiological metal ions to proteins usually caused protein aggregation and dysfunction.^17^,^18^ To further investigate the mechanism of the Ag^+^-induced loss of DNA-binding capability of CcpA, we examined the oligomerization states of CcpA before and after Ag^+^ binding using size-exclusion chromatography (SEC). In the absence of Ag^+^, WT CcpA eluted at 9.6 ml with a molecular weight (*M*_w_) of 64.9 kDa, corresponding to a dimeric form (Fig. 4a). With increasing amounts of pre-incubated Ag^+^, the intensities of the dimeric peak of CcpA decreased, whereas a new peak appeared at 8.7 ml with a *M*_w_ of 159.7 kDa, indicative of a tetrameric form of CcpA. This result implied that CcpA forms a dimer of dimers after Ag^+^ binding. A similar phenomenon was observed for the single Cys mutants of CcpA, CcpA^C216S^ and CcpA^C242S^, which also formed a tetramer after incubation with Ag^+^ (Fig. S7†). In contrast, the majority of the double mutant CcpA^2CS^ eluted at exactly the same volume as WT CcpA, even in the presence of 2 molar equivalents of Ag^+^, owing to the loss of the Ag^+^ binding capability of the protein (Fig. 4b). In line with the SEC results, native PAGE shows that Ag^+^ binding slowed down the migration rates of WT CcpA, and single mutants CcpA^C216S^ and CcpA^C242S^ in the native PAGE, which is indicative of the formation of a higher molecular weight oligomer upon Ag^+^ binding. However, Ag^+^ had no effect on the migration rate of the CcpA double mutant, CcpA^2CS^ (Fig. S8†). Taken together, the binding of Ag^+^ to CcpA induces its tetramerization, which is possibly attributable to the loss of DNA binding capability.

**Fig. 5:**
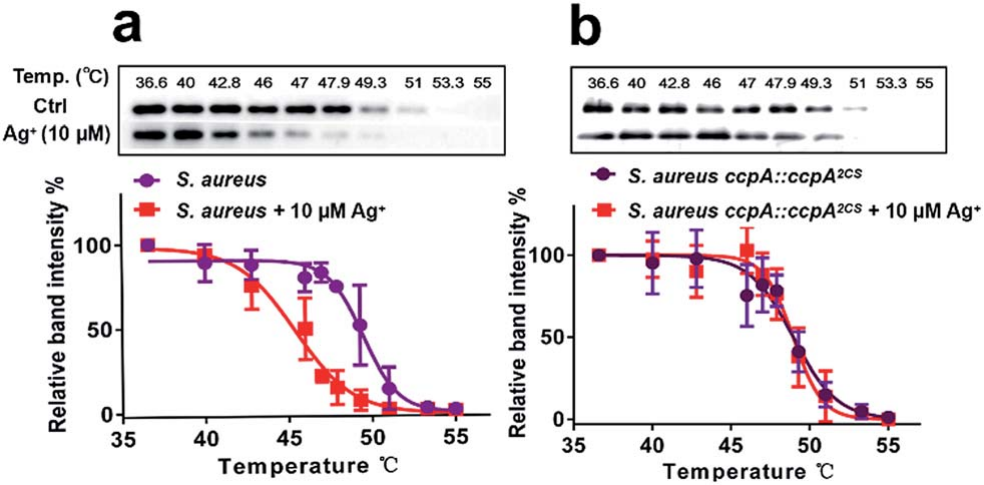
Cellular thermal shift assay (CETSA) of wild-type *S. aureus* (a) and the *S. aureus* ccpA::ccpA^2CS^ mutant (b) with or without Ag^+^ treatment. The soluble fractions of the intracellular CcpA or CcpA^2CS^ protein were quantified by a western-blot. The band intensities at different temperatures are normalized to that at 36.6 °C. All experiments were performed in triplicate.

Next, we investigated whether CcpA binds Ag^+^*in vivo* using the cellular thermal shift assay (CETSA), a method based on the change in protein thermal stability upon ligand binding for studies of the target engagement of drug candidates in a cellular condition.^19^,^20^ As shown in Fig. 5a, supplementations of 10 μM Ag^+^ to the bacterial culture resulted in the apparent aggregation temperature (*T*_agg_) of the intracellular WT CcpA shifting from 49.5 °C to 45.4 °C, indicating that Ag^+^ binds to CcpA *in vivo* and such a binding destabilizes the protein. A similar result was obtained when using purified CcpA protein (Fig. S9a†). Since Ag^+^ binds to the two Cys residues of CcpA, it is plausible to hypothesize that Ag^+^ would not change the thermal stability of the double-cysteine mutant CcpA^2CS^ due to the loss of Ag^+^ binding sites. To verify this, a CcpA gene mutant of *S. aureus*, the Newman strain, was constructed, in which the WT CcpA gene was replaced by a double cysteine mutant gene CcpA^2CS^ (denoted as *S. aureus ccpA::ccpA^2CS^*) and a similar CETSA was performed with the mutant strain. As expected, Ag^+^ treatment did not alter the intracellular CcpA thermal stability in the CcpA mutant strain (Fig. 5b). Similarly, the purified CcpA double mutant CcpA^2CS^ protein had the same thermal denaturation curves in the absence and presence of Ag^+^ (Fig. S9b†). Collectively, we demonstrated that Ag^+^ binds to CcpA intracellularly *via* its two Cys residues.

**Fig. 6:**
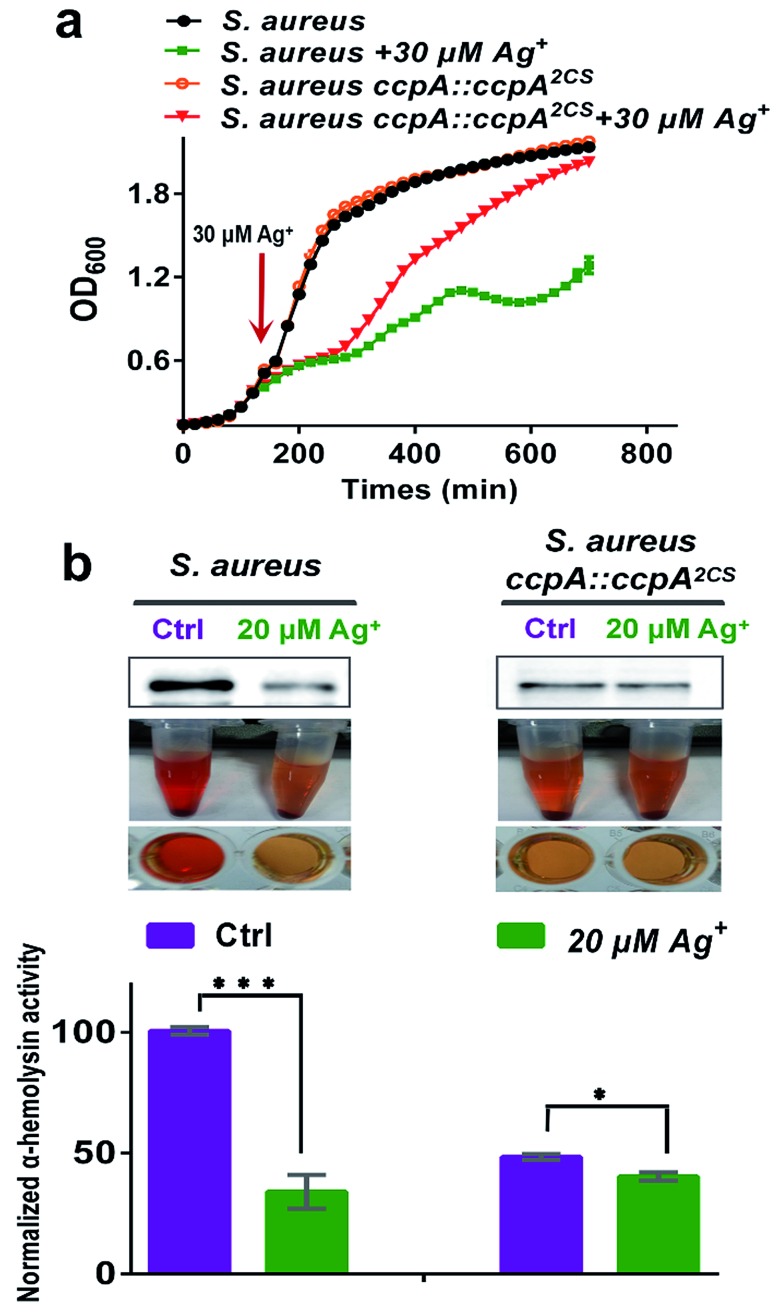
(a) The inhibition effect of Ag^+^ on the bacterial growth of the wild-type *S. aureus* and ccpA::ccpA^2CS^ mutant strains. The OD_600_ was recorded at 20 min intervals, and 30 μM Ag^+^ was added when OD_600_ reached 0.6. The secreted α-hemolysin in the supernatant was normalized to OD_600_. (b) Rabbit erythrocyte lysis activities of *S. aureus* and the *S. aureus* ccpA::ccpA^2CS^ mutant with or without 20 μM Ag^+^. Ag^+^ was added at the beginning of the bacterial culture. All experiments were performed in triplicate. Results are shown as mean ± sd. The haemolytic activities of the wild-type strain without Ag^+^ treatment are used as a control and the mean value is set at 100%. The activities in other groups are normalized to the control. The statistical difference is determined by the two-tailed Student’s *t*-test.

CcpA is the major gene regulator of central metabolism in *S. aureus* and the *CcpA* gene knockout was found to retard bacterial growth.^9^ Since Ag^+^ binds to CcpA and abolishes its function in *S. aureus*, this prompted us to investigate whether Ag^+^ affects *S. aureus* growth. We examined the bacterial growth of both the WT *S. aureus* and *ccpA::ccpA^2CS^* mutant strains upon supplementation of 30 μM Ag^+^ into the cultures at the exponential-growth phase (OD_600_ = 0.6). As shown in Fig. 6a, both the WT and *ccpA::ccpA^2CS^* mutant of *S. aureus* display nearly identical growth curves in the absence of Ag^+^. Both cultures exhibit typical S-shaped growth curves and enter a stationary-growth phase after around 250 min, indicating that *ccpA::ccpA^2CS^* did not affect *S. aureus* growth significantly. In contrast, the growth rates of both the WT and *ccpA::ccpA^2CS^* mutant of *S. aureus* were remarkably inhibited after the addition of Ag^+^ until 300 min. However, a clearly different behavior was observed for the WT and mutant cultures after 300 min, with the WT culture displaying a slower growth, yielding cell density which significantly lagged behind that of the *ccpA::ccpA^2CS^* mutant culture. After 700 min, the mutant culture with the addition of Ag^+^ reached almost the same OD_600_ value as the control group, whereas the WT culture with the addition of Ag^+^ only reached approximately 60% of the OD_600_ value of the control group. The results indicated that the *S. aureus ccpA::ccpA^2CS^* mutant is less sensitive to Ag^+^ than the WT. In line with this, the IC_50_ values of Ag^+^ for the WT and *ccpA::ccpA^2CS^* mutant of *S. aureus* were calculated to be 79.8 ± 1.1 μM and 94.1 ± 0.8 μM, respectively (Fig. S10†).

Besides regulation of carbon catabolite repression, CcpA also exerts a critical role in *S. aureus* virulence factor secretion and biofilm formation.^6^,^7^,^9^ It is reasonable to postulate that Ag^+^ would interfere with these physiological processes of *S. aureus* by targeting CcpA. Therefore, the effect of Ag^+^ on the virulence factor secretion and biofilm formation in both the WT and *ccpA::ccpA^2CS^* mutant *S. aureus* strains was investigated. The promoter region of the *hla* gene (encoding α-hemolysin) in *S. aureus* contains the cre sequence that could be recognized by CcpA. Previous studies demonstrated that the *hla* transcription level was markedly down-regulated in the *ccpA* knockout *S. aureus* strain.^21^ Indeed, secreted α-hemolysin and rabbit erythrocyte lysis activity of a *S. aureus* mutant strain with the *CcpA* gene knockout (denoted as *S. aureus* Δ*ccpA*) is almost undetectable. On the other hand, the *S. aureus ccpA::ccpA^2CS^* mutant exhibited a significant decrease on secreted α-hemolysin and retained approximately 50% erythrocyte lysis activity compared to the WT strain, implying that the two Cys residues have a potential role in the regulation of α-hemolysin expression (Fig. S11†). Upon supplementation of 20 μM Ag^+^, a western-blot showed a significant decrease in secreted α-hemolysin in WT *S. aureus*. While in the ccpA::ccpA^2CS^ mutant strain, Ag^+^ caused no obvious change in the secreted α-hemolysin. Consistently, the rabbit erythrocyte lysis activity of WT *S. aureus* decreased dramatically by 60% after Ag^+^ treatment. In contrast, the treatment of Ag^+^ led to much smaller decrease on the lysis activity of the ccpA::ccpA^2CS^ mutant strain, which still exhibited 85% activity compared to the control group (Fig. 6b and c). The results are consistent with qPCR data, which demonstrated that attenuation of the transcription level of CcpA regulated genes (*pckA* and *hla*) was much higher in the WT strain than that in the ccpA::ccpA^2CS^ mutant strain (Fig. S12†). Similarly, bacterial biofilm formation is inhibited by Ag^+^ to a lesser extent in the ccpA::ccpA^2CS^ mutant than in the WT strain (Fig. S13†). It is noteworthy that regulation of α-hemolysin expression and biofilm formation in *S. aureus* are complicated. For example, α-hemolysin expression is affected by multiple regulatory networks, including the global regulators SarA and MgrA.^22^,^23^ It is possible that these regulatory networks were also perturbed upon Ag^+^ treatment, which could at least partially explain the decrease of erythrocyte lysis activity observed in the ccpA::ccpA^2CS^ mutant strain upon Ag^+^ treatment. Nevertheless, the evident discrepancy observed between the ccpA::ccpA^2CS^ mutant and WT *S. aureus* upon Ag^+^ treatment confirms that CcpA indeed serves as one of the targets of Ag^+^*in vivo*.

**Fig. 7:**
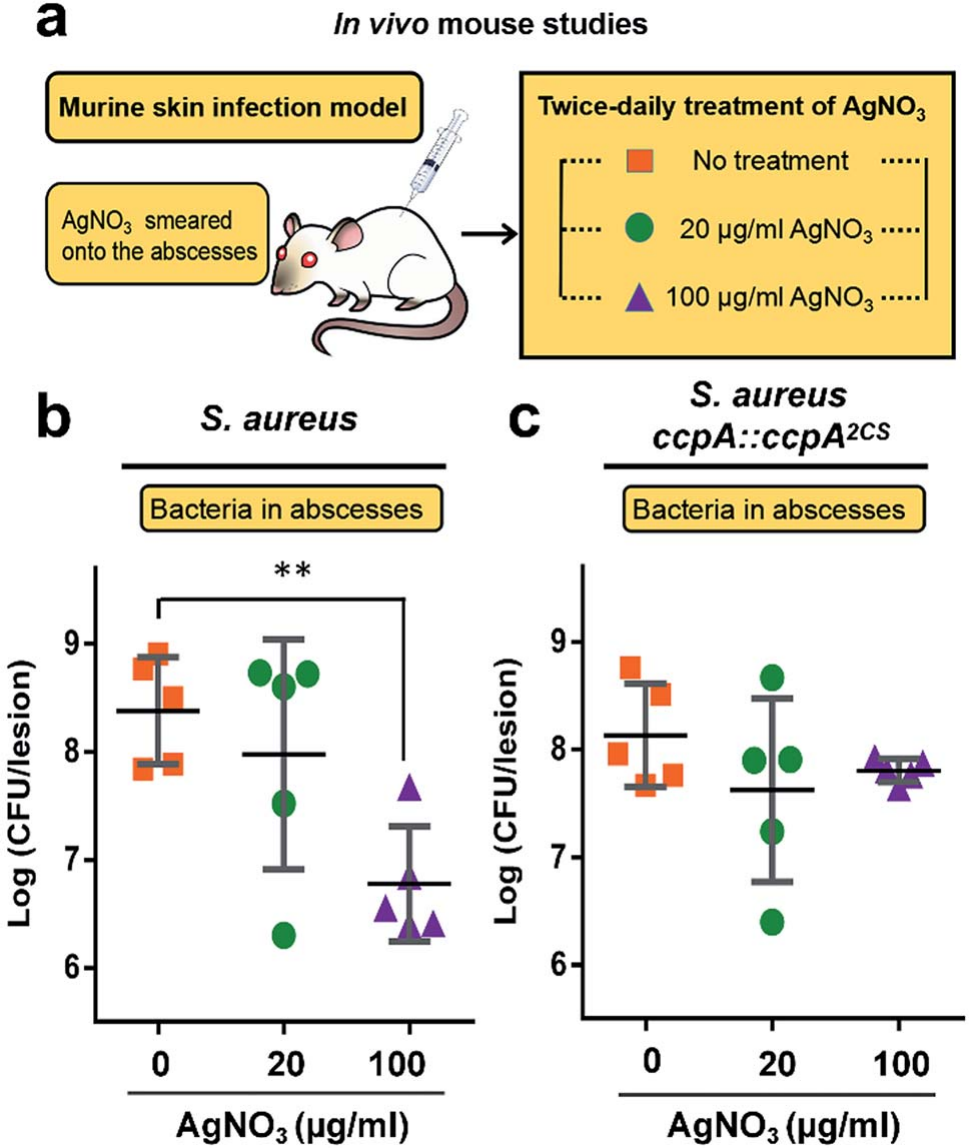
Murine skin infection model (a) to investigate the sensitivity of *S. aureus* strains to Ag^+^ treatment. The bacterial load of local abscesses induced by wild-type *S. aureus* (b) or the ccpA::ccpA^2CS^ mutant (c) was enumerated in control or AgNO_3_ treatment groups (20 μg ml^–1^ and 100 μg ml^–1^). The log CFU values are presented as the mean ± sd. The statistical difference is determined by the Mann–Whitney *U* test.

All of the results demonstrated that the WT *S. aureus* strain was more sensitive to Ag^+^ treatment than the ccpA::ccpA^2CS^ mutant strain due to Ag^+^ binding to the two cysteine residues. To further confirm this, the severity of a *S. aureus* infection was compared between the WT and ccpA::ccpA^2CS^ mutant in a murine model. The details are described in the ESI.† In brief, groups of female BALB/c mice were inoculated with *S. aureus* WT or ccpA::ccpA^2CS^ mutant strains to develop abscesses on the skin. Twice-daily treatment of AgNO_3_ with different concentrations (20 μg ml^–1^ and 100 μg ml^–1^) was applied onto the abscesses. The skin abscesses were excised 64 h post-infection, homogenized and serially diluted for CFU quantification. As shown in Fig. 7, the control groups infected with the WT or ccpA::ccpA^2CS^ mutant had similar viable bacterial counts, with log CFU mean values of 8.4 and 8.1 respectively, indicating that double Cys mutation does not significantly perturb *S. aureus* viability in a murine model. A low dosage of AgNO_3_ treatment (20 μg ml^–1^) did not change the bacterial loads in both the WT and ccpA::ccpA^2CS^ mutant infected groups. However, a significant difference was observed when the two infected groups were treated with a high dosage of AgNO_3_ (100 μg ml^–1^). In the WT *S. aureus* infected group, the viable bacterial counts dropped significantly compared to the control group. In contrast, the bacteria counts were almost the same in the ccpA::ccpA^2CS^ mutant infected groups upon a high dosage Ag^+^ treatment, confirming that WT *S. aureus* is more sensitive to Ag^+^ than the ccpA::ccpA^2CS^ mutant. Intriguingly, a significant difference on the dermonecrosis of the skin abscess was observed for the mice infected with two different bacterial strains (Fig. S14†). The mice infected with wild-type *S. aureus* had much more severe dermonecrosis than those infected with the ccpA::ccpA^2CS^ mutant strain. Given that α-hemolysin is the major contributor to necrotic lesions,^24^ the observation is consistent with the results that the ccpA::ccpA^2CS^ mutant had a lower α-hemolysin level than the wild-type strain.

## Conclusions

CcpA is one of the important global DNA regulators of Gram-positive bacteria. Recent transcriptome and proteome analyses revealed that CcpA has broad effects on gene expression in *S. aureus*, even in the absence of glucose.^25^ Particularly, a *CcpA* gene knockout abrogates biofilm formation and virulence factor expression in *S. aureus*, which remarkably decreases bacterial pathogenesis. We show clearly that Ag^+^ binds to CcpA *via* the two Cys residues both *in vitro* and *in vivo*, leading to the disruption of protein functions, thus attenuating bacterial growth, bacterial toxin expression and biofilm formation. Importantly, we demonstrated that WT *S. aureus* was more sensitive to Ag^+^ treatment than the ccpA::ccpA^2CS^ mutant in a murine skin infection model. The results herein confirm *Sa*CcpA as an intracellular target for Ag^+^. It should be noted that metal-based drugs are usually multi-targeted.^26^ Although it is commonly believed that the antimicrobial activity of silver is due to its interaction with thiol groups in enzymes and proteins, other cellular components are likely to be involved.^27^–^29^ Therefore, identification of Ag-binding proteins at a proteome-wide scale may allow extensive exploration of silver targets to advance our understanding on the bactericidal effects of silver.^30^–^32^ However, the physiological function of the two cysteine residues in *Sa*CcpA remains unclear. It has been reported previously that several DNA regulators of *S. aureus* use the cysteine-based oxidation sensing pathway for regulatory functions.^33^–^35^ Whether the two cysteines in *Sa*CcpA are also involved in oxidative sensing may warrant further studies.

## Live subject statement

The animal studies strictly followed the recommendations in “Guide for the Care and Use of Laboratory Animals” published by the National Institutes of Health. The protocols were approved by the Committee on the Use of Live Animals in Teaching and Research (CULATR) and the University of Hong Kong (Permit no. 4008-16).

## Conflicts of interest

The authors declare that there is no conflict of interest.

## Supplementary Material

Supplementary information
