# Supplementary material for: Identification of catabolite control protein A from Staphylococcus aureus as a target of silver ions
Source: Chem Sci. 2017 Oct 2;8(12):8061–6. doi: 10.1039/c7sc02251d (PMC5855135; doi:10.1039/c7sc02251d)
Supplement: Supplementary file 1 [file SC-008-C7SC02251D-s001.pdf]

Electronic Supplementary Material (ESI) for Chemical Science.
This journal is © The Royal Society of Chemistry 2017

Electronic Supplementary Information

Identification of Catabolite Control Protein A from *Staphylococcus aureus* as a Target of Silver Ions

Xiangwen Liao,[ab] Fang Yang,[a] Ruming Wang,[cd] Xiaojun He,[a] Hongyan Li,[c] Richard Y T Kao,[d] Wei Xia*[a] and Hongzhe Sun*[ac]

[a] MOE Key Laboratory of Bioinorganic and Synthetic Chemistry, School of Chemistry, Sun Yat-sen University, Guangzhou, China, 510275;

[b] Hunan Provincial Key Laboratory for Ethnic Dong Medicine Research, Hunan University of Medicine, Huaihua, China, 418000;

[c] Department of Chemistry, The University of Hong Kong, Pokfulam Road, Hong Kong, P. R. China.

[d] Department of Microbiology and State Key Laboratory for Emerging Infectious Diseases, The University of Hong Kong, Hong Kong, P. R. China.

*Correspondence and request materials should be addressed to W.X (e-mail: xiawei5@mail.sysu.edu.cn) or H.S. (e-mail: hsun@hku.hk)

Supplementary Methods and Figures

Strains, plasmids and primers. Strains, plasmids and primers are listed in Table S1. The *Escherichia coli* (*E. coli*) XL1-Blue and BL21(DE3) strains were cultured in LB medium with required antibiotics. *Staphylococcus aureus* (*S. aureus*) strains were cultured in Tryptic Soy Broth (TSB) medium (17 g Tryptone, 3 g Phytone, 5 g NaCl, 2.5 g dipotassium phosphate (K_2HPO_4) and 2.5 g glucose per liter) or on Tryptic Soy Agar (TSA). Custom polyclonal antisera to *S. aureus* CcpA were prepared by immunizing rabbit with purified CcpA protein (Huabio, China). The rabbit polyclonal antibodies were purified from antisera by Protein A sefinose (Sangon, China).

Cloning, expression and purification of CcpA and mutant proteins. *ccpA* gene was amplified by PCR using *S. aureus* Newman chromosomal DNA as a template with the primers listed in Table 1 containing NdeI and BamHI restriction site at 5'- and 3'-end, respectively. The corresponding amplified product was digested with NdeI and BamHI and ligated into the pET47b plasmid, which has been digested with the same restriction enzymes. The generated pET47b-*ccpA* plasmid was transformed into BL21(DE3) cell for protein expression. Generally, overnight culture of BL21(DE3) cell that harbored the pET47b-*ccpA* plasmid was diluted by 1:100 to fresh LB medium supplemented with 50 μg/mL Kanamycin and grown for 3 hrs aerobically at 37 ^{o}C until OD_{600} reached 0.6. Protein expression was induced by addition of IPTG to a final concentration of 0.5 mM and bacteria were further incubated at 25 ^{o}C for overnight. The bacteria were harvested by centrifugation at 5000×g for 20 min at 4 ^{o}C and the cell pellet was resuspended with 20 mM Tris-HCl, 150 mM NaCl (pH=7.4) buffer. Cell pellet was lysed by sonication at 4 ^{o}C with 1 mM PMSF as protease inhibitor. The lysates were centrifuged at 15000× g for 10 min and the supernatant was collected. The supernatant was then dialyzed against 50 mM Tris-HCl, 1 mM DTT (pH=8.0) buffer and subsequently applied to a 5 mL HiTrap Q column (GE Healthcare). Elution was performed with a linear gradient from 0 to 500 mM NaCl in 20 mM Tri-HCl buffer, 1 mM DTT, pH 8.0. The eluted protein was pooled and further purified by Hiload 16/60 Superdex 75 column equilibrated with Tris-HCl buffer (20 mM Tris-HCl, 300 mM NaCl, 1 mM DTT, pH 7.4). Purified protein was buffer-exchanged into Tris-HNO_3 buffer (50 mM Tris-HNO_3, 150 mM $NaNO_3$, pH 7.4) by HiTrap desalting column (GE healthcare) to remove DTT and chloride ion immediately before use. Plasmids for CcpA^{C216S}, CcpA^{C242S}, CcpA2^{CS} mutant expression were generated via site-directed mutagenesis using Phusion high fidelity DNA polymerase. Wild-type pET47b-*ccpA* plasmid was used as DNA template. The primers used for mutants are listed in Table 1. The expression and purification of CcpA mutants were similar to wild-type CcpA.

Cloning, expression and purification of phosphorylated HPr. The *hpr* and *hprK* genes were PCR amplified by using *S. aureus* Newman chromosomal DNA as a template with the primers listed in Table 1 containing EcoRI and HindIII restriction site at 5'- and 3'-end, respectively. The amplified DNA was digested with EcoRI and HindIII and cloned into pET47b or pET32a plasmid. The generated pET47b-*hpr* and pET32a-*hprK* plasmid was transformed into BL21(DE3) cell for protein expression. His_6-tagged protein expression was induced by 0.5 mM IPTG in Luria broth at 25 °C and purified with Ni-NTA Resin.

The phosphorylation assays were performed in the presence of His_6-HPr and His_6-HPrK in phosphorylation buffer (10 mM Tris-HCl, 50 mM KCl, 5 mM $MgCl_2$, 1 mM DTT, and 10%

glycerol, pH 7.4). The reactions were carried out after adding 10 mM ATP at 37°C for 30 min. Subsequently, the phosphorylated His_6-HPr (HPr-P) was incubated with His_6-prescisson protease to remove the His_6-tag at 4 °C overnight. Subsequently, the His_6-prescisson protease and uncleaved HPr-P was removed by Ni-NTA column. The eluted protein was pooled and further purified by Hiload 16/60 Superdex 75 column equilibrated with Tris-HCl buffer (20 mM Tris-HCl, 300 mM NaCl, 1 mM DTT, pH 7.4).

Electrophoretic mobility shift assays (EMSA). The DNA probes containing the promoter regions of *pckA*, *hla* and *proC* for electrophoretic mobility shift assays (EMSA) were generated by PCR using *S. aureus* Newman chromosomal DNA as a template with primer pairs listed in Table 1. For CcpA-DNA binding assay, the EMSA reaction mixtures were set up in a final volume of 10 µl, which contained 35 nM DNA probe, various concentrations of the purified CcpA and $CcpA^{2CS}$ proteins (0, 75, 150, 300, 450, 600 and 700 nM). For Ag^+ perturbation assay, 500 nM CcpA or $CcpA^{2CS}$ were incubated with different molar equivalents of Ag^+ (0, 0.4, 0.8, 1.2, 2 and 3) for 30 min at room temperature in binding buffer (25 mM Tris-HNO_3, 80 mM $NaNO_3$, 35 mM KNO_3, 10 mM $Mg(NO_3)_2$, 0.1% Triton X-100 and 10% glycerol, pH 7.5). To investigate the effect of Ag^+ on the formation of CcpA-DNA-(HPr-P) ternary complex, 500 nM CcpA was incubated with increasing concentration of HPr-P (2, 4, 6, 8 and 10 molar equivalents) in the presence of 2 molar equivalents of Ag^+ for 30 min at room temperature in binding buffer. Subsequently, 10 µl of the reaction mixture were loaded onto a native 6% (w/v) polyacrylamide TBE Gel and electrophoresed in 0.5×TBE (v/v) buffer for 45 min at 200 $V.cm^{-1}$. Gels were stained in a 10,000-fold diluted Gene-Finder nucleic acid staining solution (Xiamen Zhishan Ltd, China) for 5 min. The DNA bands were visualized with blue light transilluminator (Syngene).

Ag^+ content determination of CcpA and mutant proteins. The metal contents of protein were determined by inductively coupled plasma mass spectrometry (ICP-MS). All ICP-MS experiments were conducted on a Thermo Scientific iCAP Q ICP-MS spectrometer. Each sample was quantified in triplicate and the average value was used. Around 3 molar equivalents of Ag^+ were added into approximate 100 µM CcpA, $CcpA^{C242S}$, $CcpA^{C216S}$ and $CcpA^{2CS}$ in Tris-HNO_3 buffer. After incubation for 30 min at room temperature, excess amounts of Ag^+ were removed by HiTrap desalting column (GE healthcare). The eluted protein concentration was measured by bicinchoninic acid (BCA) assay.

Ellman assay. Ellman assay was carried out in Tris-HNO_3 buffer. Different molar equivalents of Ag^+ were added into 50 µM CcpA or mutants and incubated for 20 min at room temperature. Excess amounts of 5, 5-dithio-bis-(2-nitrobenzoic acid) (DTNB) were added with a final concentration of 160 µM. After further incubation for 20 min, UV absorbance of each sample at 412 nm was measured by UV-vis spectroscopy. The absorbance at 412 nm was plotted against Ag^+/protein ratio.

Isothermal titration calorimetry. Isothermal titration calorimetry (ITC) experiments were performed on a Malvern MicroCal iTC200 at 25 °C. The CcpA, $CcpA^{C242S}$ and $CcpA^{2CS}$ were prepared in Tris-HNO_3 buffer with a final concentration of 50 µM. The Ag^+ titrant was prepared

by dissolving $AgNO_3$ in Tris-HNO_3 buffer with concentration of 1 mM. Typically, 40 μl of $AgNO_3$ titrants were titrated into 200 μl protein sample with 150 s interval between each injection. The signals of $AgNO_3$ titration into Tris-HNO_3 buffer were recorded as background control. All ITC data were analyzed using the Origin software provided and fitted by one set of site binding model.

Bio-layer inferometry assay (BLI). Interactions between purified CcpA and the biotin labeled promoter region of *pckA* were measured using biolayer inferometry (BLI) on an Octet Red96 system (ForteBio). Binding experiments were performed at 37 °C in binding buffer (25 mM Tris-HNO_3, 80 mM $NaNO_3$, 35 mM KNO_3, 10 mM $Mg(NO_3)_2$, 0.1% Triton X-100, 10% glycerol, 0.1% BSA and 0.01% Tween 20, pH 7.5). Around 300 nM of biotinylated DNA probe was captured on pre-immobilized streptavidin Dip and Read sensor heads for 3 min. DNA-immobilized sensor heads were subsequently incubated in binding buffer for 30 s to reach equilibrium. The sensor was subsequently incubated in binding buffer containing various concentrations of purified CcpA or $CcpA^{2CS}$ (100 nM to 6.25 nM in two-fold dilutions) with or without silver ions and the association of the protein with promoter was measured over 180 seconds. Dissociation was subsequently monitored over 180 seconds in binding buffer without protein. All data were normalized and analyzed using the global fit binding model over all concentrations using the software provide by Fortebio to derive k_{on}, k_{off} and K_D values.

Protein oligomerization state analysis. The oligomerization states of CcpA, $CcpA^{C242S}$, $CcpA^{C216S}$ and $CcpA^{2CS}$ were analyzed by size-exclusion chromatography. CcpA, $CcpA^{C242S}$, $CcpA^{C216S}$ and $CcpA^{2CS}$ were incubated with different molar equivalent of Ag^+ at room temperature for 30 min. The elution volumes of samples were subsequently measured on a Tricorn Superdex 75 10/300 GL column (GE Healthcare) pre-equilibrated with Tris-HNO_3 buffer. The column was calibrated with GE LMW calibration kit with the same buffer condition.

Native polyacrylamide gel electrophoresis (PAGE) assay. Native PAGE (12%) was used to analyze the migration of CcpA and corresponding mutant with or without Ag^+ treatment. The native PAGE was prepared as described previously.[1, 2] Purified protein was buffer-exchanged into Tris-HNO_3 buffer via desalting column and then divided into two groups, one group was incubated with 2 molar equivalents of Ag^+ for 30 min. To investigate whether Ag^+ perturbed CcpA-DNA-(HPr-P) ternary complex formation, around 7 μM CcpA was incubated with 5 μM HPr-P and 2 μM *pckA* DNA probe in the absence or presence of 2 molar equivalents Ag^+ (14 μM) for 30 min. Subsequently 4× native PAGE loading buffer (1.0 M Tris-HCl, 1% bromophenol blue and 50% glycerol) was added into the protein samples. The electrophoresis was carried out at 130V for 1 h on ice with 25 mM Tris-HCl, 250 mM glycine as running buffer. Gels were stained with Coomassie brilliant blue.

***S. aureus* mutant construction.** The allelic exchange plasmid pKOR1 was used to obtain the *S. aureus* Δ*ccpA* and *ccpA::ccpA*^{2CS} mutant as previously described.[3, 4] Primers used to generate flanking DNA fragments for allelic replacement are listed in Table S1. For *S. aureus* Δ*ccpA* mutant, a flanking DNA fragment contains only 1 kb upstream and 1 kb downstream of *ccpA* gene.

For *S. aureus ccpA::ccpA^{2CS}* mutant, a 2.9-kp flanking DNA fragment that covers the *ccpA* gene, 1 kb of its upstream and 1 kb of its downstream regions was amplified by PCR from *S. aureus* Newman chromosomal DNA and cloned into pBlusScript II SK^+ plasmid. The two cysteines C216 and C242 were mutated into serines via site-directed mutagenesis to generate the *ccpA^{2CS}* mutant flanking DNA fragment. Both of the flanking DNA fragments were subsequently amplified and cloned into pKOR1 using Gateway BP Clonase (Invitrogen) to generate the plasmid pKOR1-Δ*ccpA* and pKOR1-*ccpA*^{2CS}. The resulting plasmids were transferred via electroporation first to *S. aureus* RN4220 (a restriction minus and modification plus strain) to modify DNA, and subsequently to *S. aureus* Newman generating *S. aureus* Δ*ccpA* and *S. aureus ccpA*::*ccpA*^{2CS} mutant strains.

Allelic replacement was carried out by a two-step procedure as described previously. In the first step, plasmid was integrated into the chromosome by *S. aureus* growth at the pKOR1 plasmid non-permissive duplication condition (43 °C), and in the second step, plasmid eviction was induced by growing cells at the permissive temperature. To integrate the plasmid, 5 µl of *S. aureus* culture that harbored the pKOR1 plasmid were transferred into 5 ml of fresh TSB medium with 10 µg/ml chloramphenicol (TSB_{Cm10}) pre-warmed at 43 °C and incubated overnight at the same temperature. In the following day, the procedure was repeated and the culture was continually incubated at 43 °C in TSB_{Cm10}.. Culture aliquots were streaked on tryptic soy agar 10 µg/ml chloramphenicol (TSA_{Cm10}) and further incubated at 43 °C overnight. From the resulting plate, six colonies were picked and inoculated into 5 ml fresh TSB and incubated at 30 °C overnight to facilitate plasmid excision.

Cultures were then 10^6-fold diluted with sterile water and 100 µl aliquots were spread on TSA and incubated at 30 °C. To identify the desired allelic exchange on TSA, six colonies were inoculated in TSB and incubated at 37 °C overnight. Chromosomal DNA was purified from the cultures and *ccpA* gene was PCR-amplified and sent for DNA sequencing (Sangon, China).

Cellular thermal shift assay (CETSA). Wild-type *S. aureus* Newman and *S. aureus ccpA::ccpA^{2CS}* Newman strains were cultured in TSB medium until OD_{600} reached 1. The cultures were subsequently 1:100 diluted into fresh TSB and grown until OD_{600} reached 0.6. Approximate 10 µM $AgNO_3$ were added into the experimental group bacterial cultures and incubated at 37 °C for 2.5 hrs. The bacterial cells were then harvested by centrifugation at 5000×g for 10 min at 4 °C. Equal amounts of cell suspensions from control and experimental groups were aliquoted into PCR tubes and heated individually at increasing temperatures for 3 min followed by immediate cooling on ice. The heating procedures were repeated three times. The cells were then lysed by 3 freeze-thaw cycles using liquid nitrogen. The cell lysates were centrifuged at 15,000×g for 10 min at 4 °C to pellet the denatured, precipitated proteins. The soluble proteins in the supernatants were analyzed by gel electrophoresis followed by immuno-blotting using CcpA antibodies.

For the thermal denaturation curves of purified proteins, purified CcpA and CcpA^{2CS} protein was buffer-exchange into Tris-HNO_3 buffer (50 mM, 150 mM $NaNO_3$, pH 7.4) using desalting column (GE healthcare). Subsequently 2 molar equivalents of Ag^+ were added immediately into experimental group of protein samples. After incubation for 30 minute, equal amounts of protein solution both from control and experimental samples were aliquoted into PCR tubes and heated individually at increasing temperatures for 3 min three times followed by immediate cooling on ice. Denatured protein pellets were removed by centrifugation at 15,000×g for 10 min at 4 °C. The

soluble fractions in the supernatants were analyzed by SDS-PAGE.

Measurement of gene expression by Quantitative Real Time PCR. The wild-type *S. aureus* Newman and *ccpA::ccpA^{2CS}* mutant strains were grown overnight in TSB medium. The next day, the cell cultures were 1:100 diluted into fresh TSB and grown until OD_{600} reached 0.6. Subsequently, the culture was treated with 10 μM $AgNO_3$ at 37 °C for 1 hour. Total RNAs from both untreated (as control) and treated group were isolated with SV total RNA isolation system (Promega) according to the manufacturer's recommendations. cDNA was generated by reverse transcription using GoScript Reverse Transcriptase (Promega). The transcription level of detected gene was subsequently determined by real-time PCR using GoTaq qPCR Master Mix kit (Promega) on a StepOnePlus Real-time PCR system (Life Technologies). The *rrsA* (16S rRNA) was used as an internal control. All of the experiments were conducted in triplicate and relative expression levels were measured using the $2^{-\Delta\Delta Ct}$ method. The mean value before Ag^+ treatment was set as 1 and data are presented as mean ± SD. The Ag^+ treated groups were normalized to that of the control groups. The following primers were used for real-time PCR: 16S rRNA-For and 16S rRNA-Rev, *hal*-For and *hla*-Rev, *pckA*-For and *pckA*-Rev (Table S1).

Effect of Ag^+ on *S. aureus* growth. Wild-type *S. aureus* and *S. aureus ccpA::ccpA^{2CS}* mutant growth curves were measured by Biotek Cytation3 plate reader using the dynamics model. Briefly, a single colony of wild-type *S. aureus* or mutant strains was picked from TSB Agar plate and cultured in TSB medium. After OD_{600} of the culture reached 1 (~1.5×10^8 CFU/ml), the culture was diluted 10 times with fresh TSB medium (OD_{600}=0.1). The bacterial cultures were then incubated at 37 °C in plate reader with orbital shaking at 220 rpm in a 24-well plate. The bacterial growth was monitored via OD_{600} values at 20-minute intervals. For the Ag^+ treatment, 30 μM of Ag^+ were added after OD_{600} reached up to 0.6.

The IC_{50} of Ag^+ against wild-type *S. aureus* and *S. aureus ccpA::ccpA^{2CS}* mutant was measured using the similar method for minimal inhibitory concentration (MIC) assay as described previously.[5, 6] Briefly, wild-type *S. aureus* Newman and *S. aureus ccpA::ccpA^{2CS}* mutant strains were cultured in TSB medium until OD_{600} reached up to 1. The bacterial cell numbers were diluted to approximately 5×10^6 CFU/ml (OD_{600}=0.05) with fresh TSB medium. Subsequently, 10 μl of Ag^+ with different concentrations were added into 190 μl of the bacterial suspension. The mixtures were put into a 96-well plate and further incubated at 37 °C for 20 hrs. The OD_{600} of each well was measured by Biotek Cytation3 plate reader. The Ag^+ inhibition curves were plotted and IC_{50} values were calculated using GrapPad Prism software.

Hemolysin activity assay. Hemolysin levels in bacterial culture supernatants were determined based on the method described.[7, 8] Briefly, rabbit blood cell was prepared by wash the defibrinated rabbit blood with BSA buffer (20 mM KH_2PO_4, 150 mM NaCl, 1 mM $MgCl_2$, 1 mg/ml BSA) twice and collected via centrifugation (2000×g, 2 min). Supernatant was obtained by centrifugation (12000×g at 4 °C, 5 min) 0.5 ml of the bacterial samples, which were grown for 9 hrs at 37 °C. Approximate 0.1 ml of supernatant was diluted to 1 ml with BSA buffer and mixed with 20 μl of defibrinated rabbit blood cell. After incubation for 25 min at 37 °C, intact blood cells were removed by centrifugation (5500×g, room temperature, 1 min) and the optical densities of the supernatants were measured at 543 nm. The secreted α-hemolysin was quantified by western

blot, 20 μl culture supernatants from the hemolytic activity assay were boiled with 6×SDS loading buffer and then applied to 10% polyacrylamide gel followed by immuno-blotting using the anti-Staphylococcal α-Toxin antibody (Sigma-Aldrich).

Effect of Ag^+ on *S. aureus* biofilm formation. Biofilm assays were performed using 24-well polystyrene flat-bottom microtiter plate as described previously with a few modifications.[9-11] Briefly, 24-well polystyrene flat-bottom microtiter plates were pre-coated with 1 ml 10% Albumin from bovine serum (Sigma) in Tris-HCl buffer (pH 9.4) and incubated for 24 hrs at 4 °C. Wild-type *S. aureus* and *S. aureus ccpA::ccpA^{2CS}* Newman mutant strains were cultured in TSB medium for 5 hrs. The cultures were then diluted 1:200 with fresh sterile TSB medium containing 0.5% glucose. After removing the 10% Albumin from bovine serum, the 24-well microtiter plate was filled with 1 ml aliquots of the bacterial culture with or without 20 μM Ag^+. The plates were incubated at 37 °C for 36 hrs. Afterwards, non-adherent bacteria were washed away by PBS for three times and the plate was dried out overnight at room temperature. Biofilm attached to microtiter plates was strained by 0.1% crystal violet solution (Sangon, China) for 15 minutes. Excess stain was removed by washing with PBS. The crystal violet attached to biofilm samples was dissolved with acetic acid. The absorbance at 595 nm was measured using Biotek Cytation3 plate reader to indicate biofilm formation.

Murine skin infection model. Overnight cultures of bacteria (*S. aureus* Newman and *S. aureus ccpA::ccpA^{2CS}*) were 1:100 diluted in fresh TSB medium and regrown till OD_{600}=0.6 at 37 °C with shaking (250 rpm). Bacterial pellets were collected and washed by PBS three times for further use. For the skin infection, mouse hair in the infection site (of area ~ 2 cm^2) was removed by Veet® (according to the manufacture instruction) one day before infection. Groups of mice (female BALB/c strain, 6~8 weeks of age, 18~22 g of weight) were developed with measurable abscesses 16 hours post subcutaneous infection with 3×10^7 CFU of bacteria in a 50 μL aliquot of PBS. Mice infected either by *S. aureus* Newman or *S. aureus ccpA::ccpA^{2CS}* were divided into three groups (n = 5 for each group) including control (vehicle), $AgNO_3$ (20 μg/ml) and $AgNO_3$ (100 μg/ml). $AgNO_3$ were mixed thoroughly in sterile cream and smeared onto the abscesses in a 50 μL aliquot. Twice-daily treatment of $AgNO_3$ was continued throughout the whole experimental course. All the mice were sacrificed 64 hours post-infection. Local abscesses were collected homogenized (Qiagen® Tissue Lyser II at a frequency of 30 Hz for 5 min). Tissue homogenates were serially diluted in PBS and then spread onto agar plates for the enumeration of bacterial load.

.

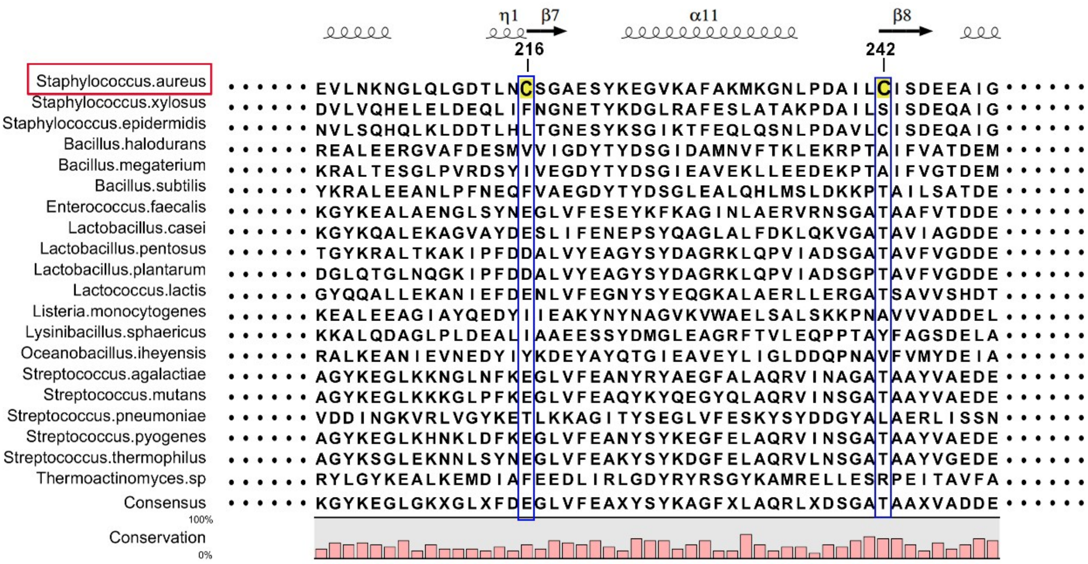

Fig. S1 Sequence alignments of CcpA homologues from Gram-positive bacteria. The two unique cysteines residues are highlighted in blue box.

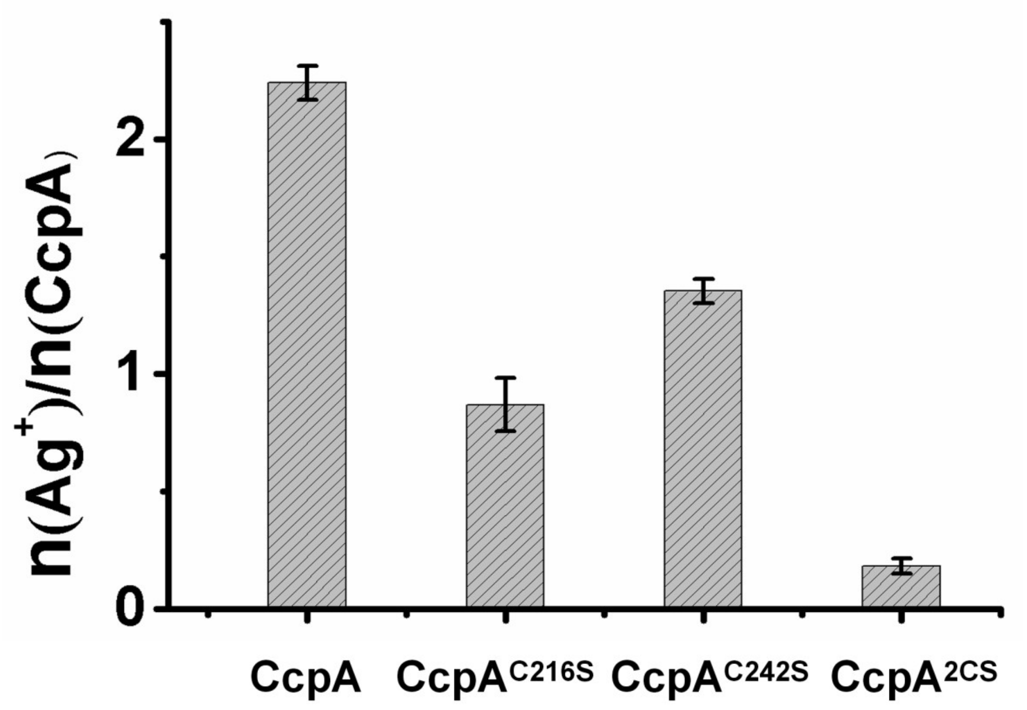

Fig. S2 Ag^{+}-binding capability of CcpA, $CcpA^{C216S}$, $CcpA^{C242S}$ and $CcpA^{2CS}$ determined by ICP-MS. All samples were pre-incubated with 3 molar equivalents of Ag^{+}. Access amounts of Ag^{+} were removed by desalting column. The bound Ag^{+} contents were determined by ICP-MS and protein concentrations were measured by BCA assay. All measurements were performed in triplicate.

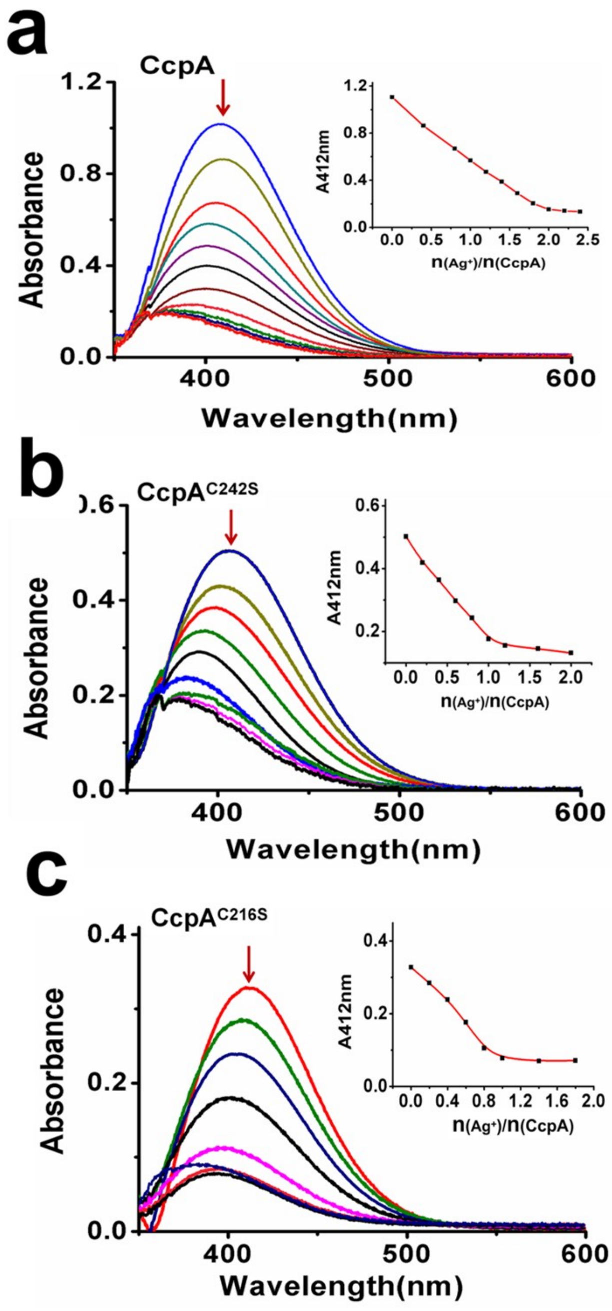

Fig. S3 Ag^{+}-binding to CcpA(a), CcpA^{C242S} (b) and CcpA^{C216S}(c) monitored by Ellman's assay. CcpA and mutants were pre-incubated with various molar equivalents of Ag^{+} as indicated. The free thiol contents in protein samples were measured by adding excess amounts of DTNB (Ellman's reagent). The absorbance at 412 nm was plotted against Ag^{+}/protein ratios.

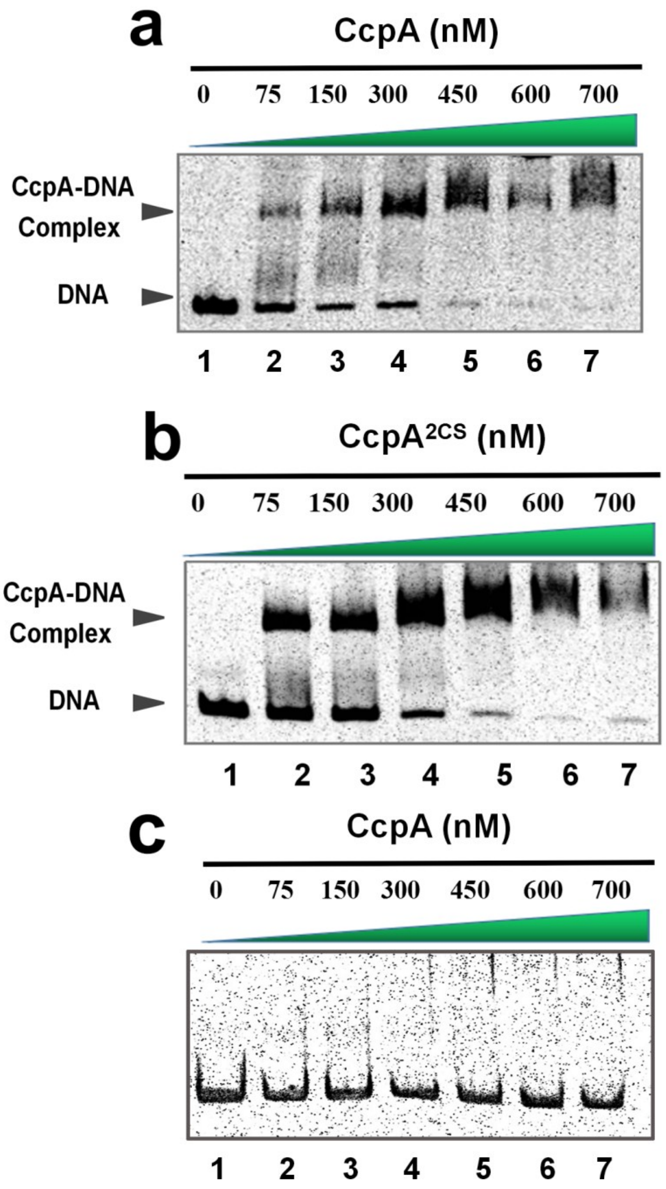

Fig. S4 Electrophoretic mobility shift assay (EMSA) of CcpA binding to the catabolite responsive elements (*cre*) of the *pckA***.** 35 nM *pckA* promoter (189 base pairs) was incubated with different concentrations of purified CcpA **(a)** or CcpA^{2CS} **(b).** As a negative control, 35 nM *proC* (encoding Δ^1-pyrroline 5-carboxylate [P5C] reductase) promoter (188 base pairs) was incubated with increasing concentrations of CcpA (**c**)**.**

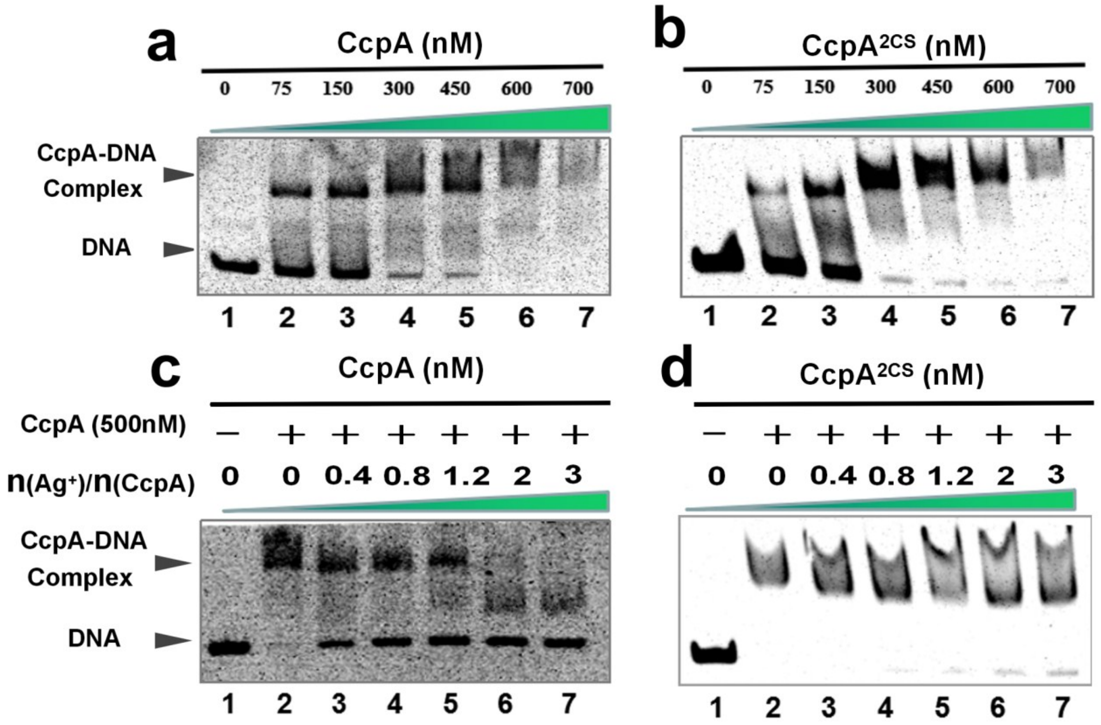

Fig. S5 Electrophoretic mobility shift assay (EMSA) of CcpA binding to the catabolite responsive elements (*cre*) of the *hla.* 35 nM *hla* promoter (330 base pairs) were incubated with gradient amounts of purified CcpA (**a**) or CcpA^{2CS} (**b**). 35 nM *hla* promoter incubated with 500 nM purified CcpA (**c**) or CcpA^{2CS} (**d**) pre-incubated with 0, 0.4, 0.8, 1.2, 2 and 3 molar equivalents of Ag^+.

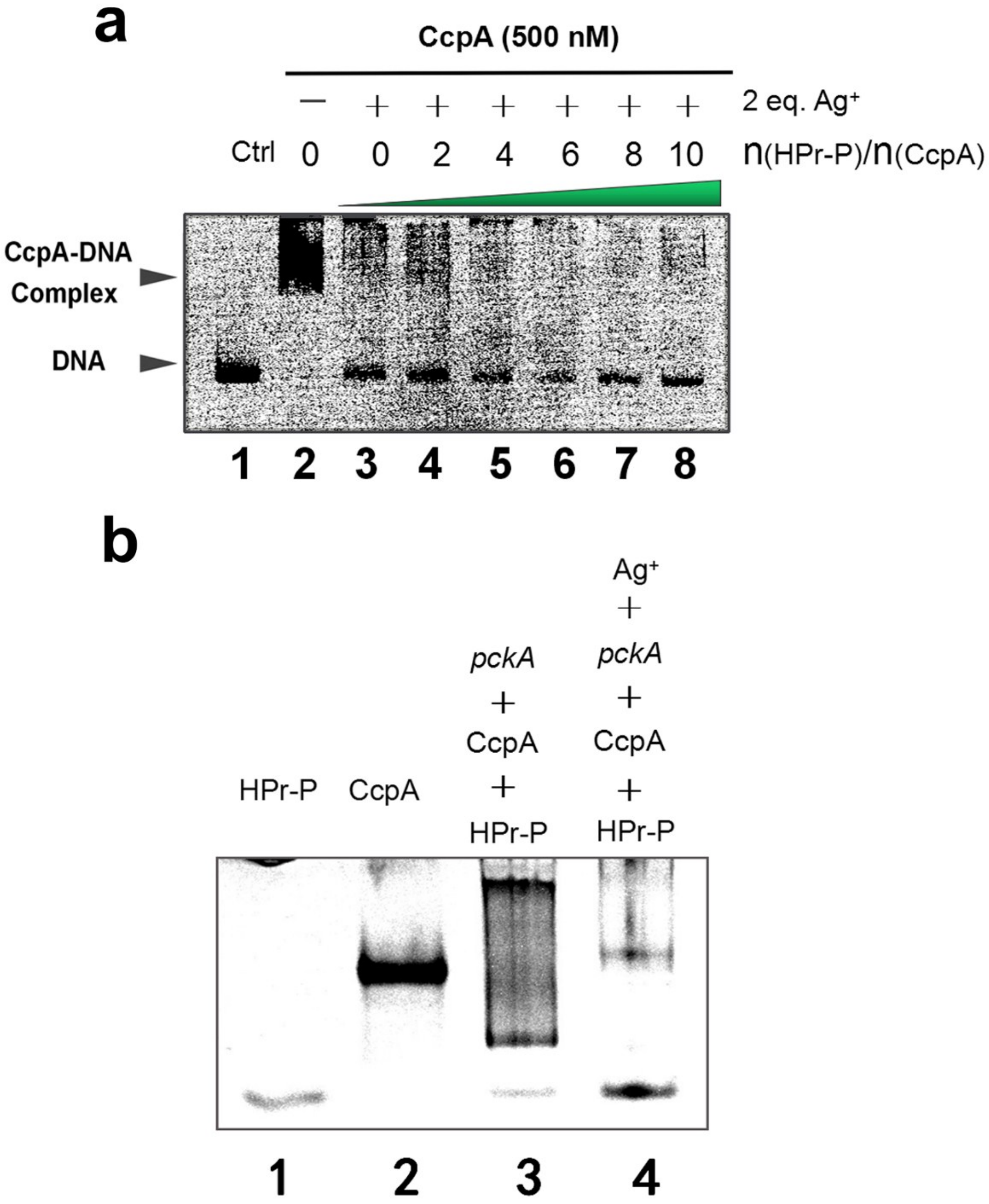

Figure S6 (a) Electrophoretic mobility shift assay (EMSA) of CcpA binding to the catabolite responsive elements (*cre*) of the *pckA* in the presence of Ag^+ and phosphorylated Hpr. 35 nM *pckA* promoter (189 base pairs) were incubated with 500 nM purified CcpA and different molar equivalents of HPr-P (2, 4, 6, 8 and 10) in the presence of 2 molar equivalents of Ag^+. **(b)** Native polyacrylamide gel electrophoresis (PAGE) analysis of CcpA/HPr-P complex. Around 7 µM CcpA were incubated with 5 µM HPr-P and 2 µM *pckA* DNA probe in the absence or presence of 2 molar equivalents Ag^+ (14 µM) for 30 min before native PAGE analysis.

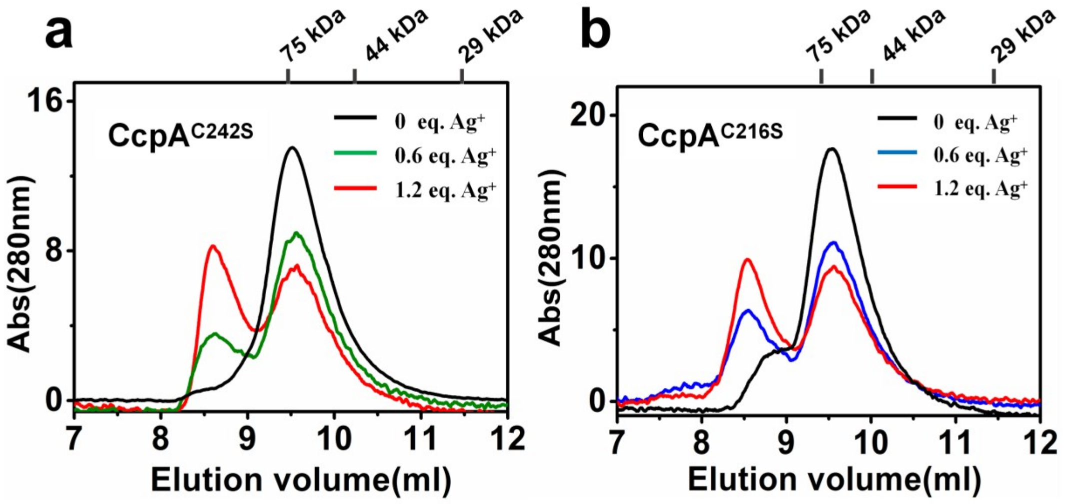

Fig. S7 The effects of Ag^{+} binding on the oligomeric states of $CcpA^{C242S}$ (a) and $CcpA^{C216S}$ (b). Size-exclusion chromatography analysis of the $CcpA^{C242S}$ and $CcpA^{C216S}$ incubated with 0, 0.6 and 1.2 molar equivalents of Ag^{+}.

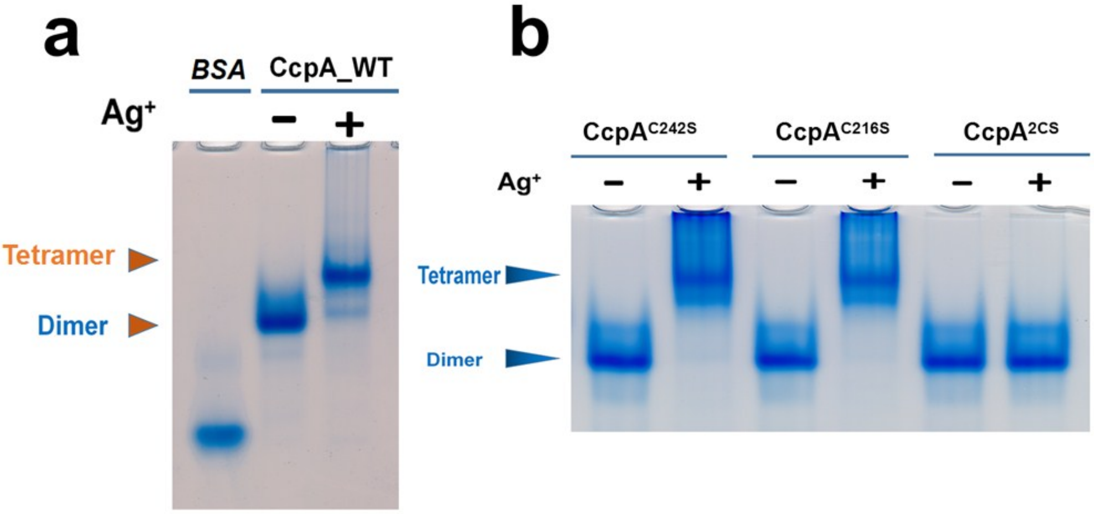

Fig. S8 Native polyacrylamide gel electrophoresis (Native PAGE) to monitor the oligomeric state changes of CcpA **(a)** and mutants **(b)** upon Ag^+ binding. Purified CcpA and mutants incubated with 2 molar equivalents of Ag^+ were applied to native PAGE for analysis.

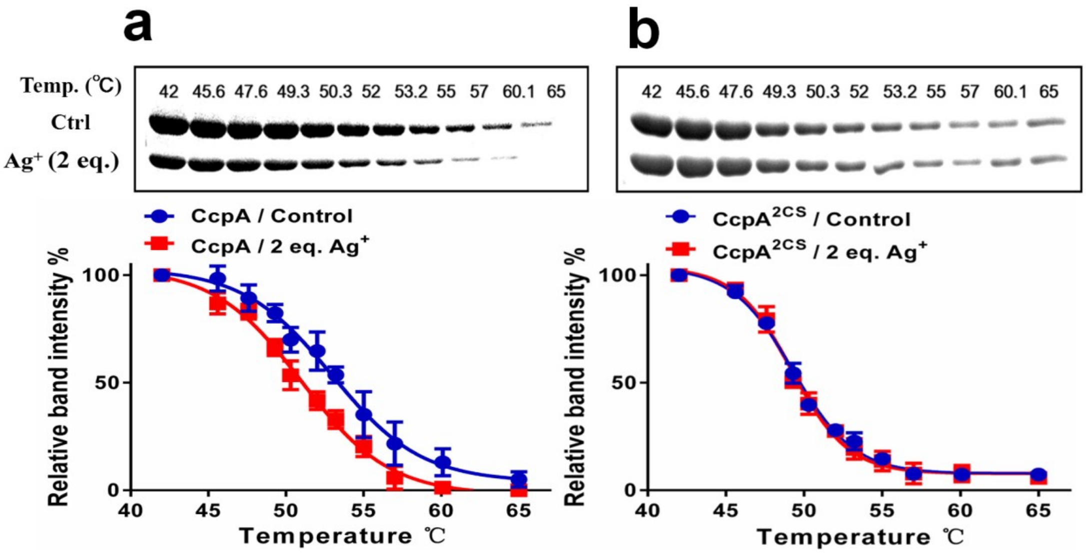

Fig. S9 Thermal denaturation curves of purified proteins. Purified CcpA **(a)** and CcpA^{2CS} protein **(b)** with or without Ag^{+} treatment were heated at a gradient temperature. The soluble fractions of the protein were analyzed by SDS-PAGE. The band intensities at different temperatures are normalized to that at 42 ^{o}C.

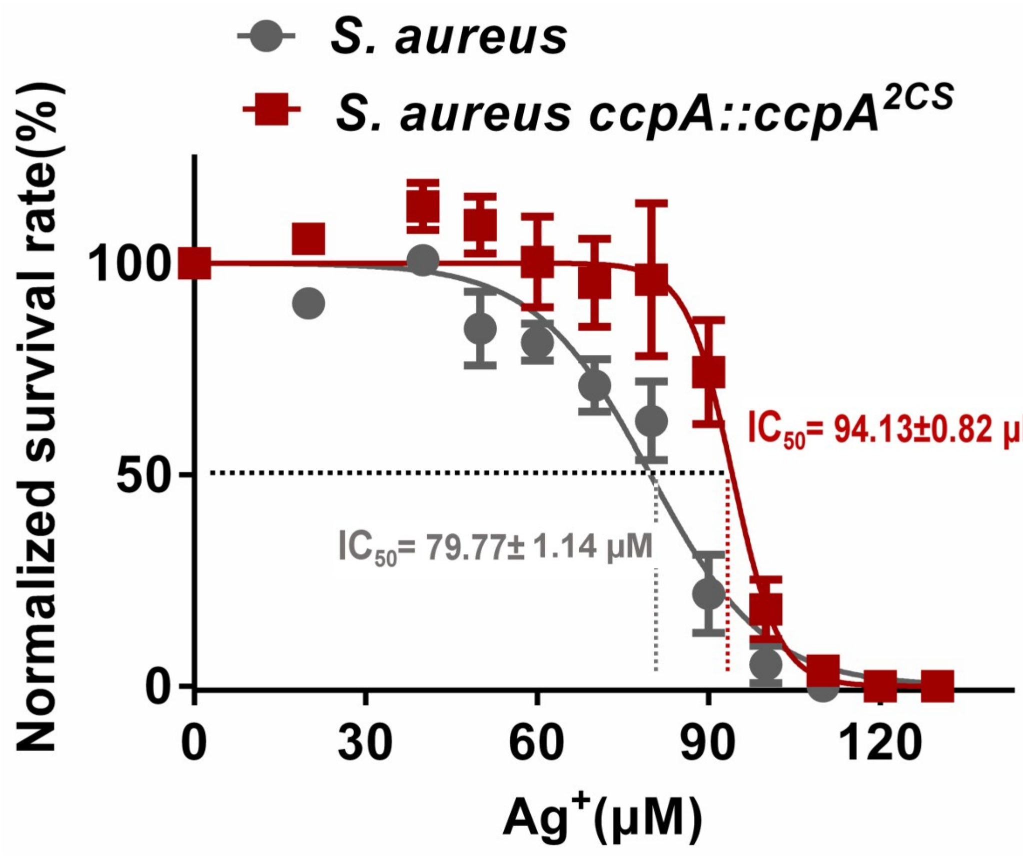

Fig. S10 Measurement of IC_{50} values of Ag^+ against *S. aureus* and *S. aureus ccpA::ccpA^{2CS}* mutant. The experiment was performed in a 96-well plate and the OD_{600} of each well was measured by Biotek Cytation3 plate reader. The Ag^+ inhibition curves were plotted and IC_{50} values were calculated using GrapPad Prism software.

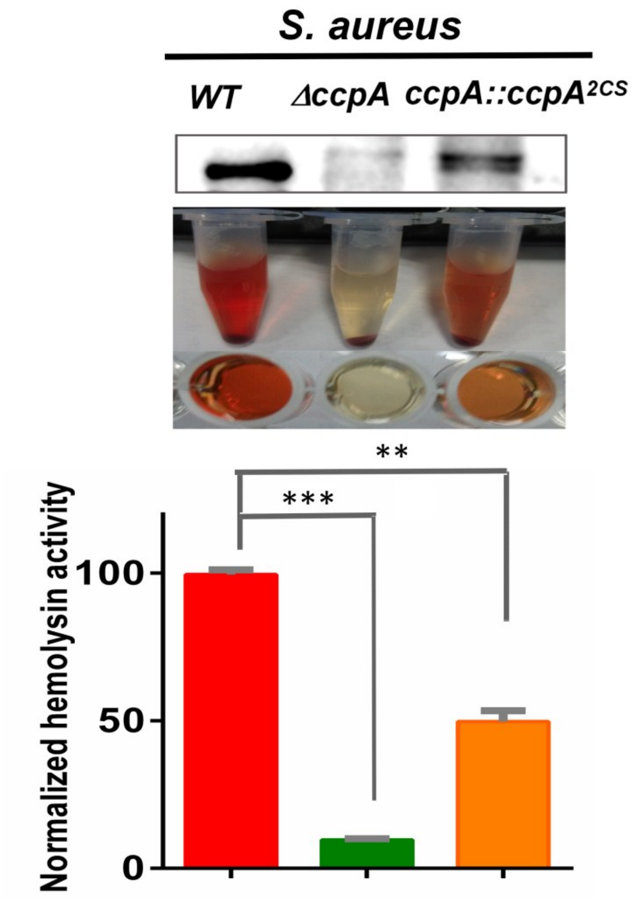

Fig. S11 The erythrocyte lysis activities and α-hemolysin Western blot analysis of *S. aureus*, *S. aureus ΔccpA* and *S. aureus ccpA::ccpA*^{2CS} mutant. The activities were measured by adding 100 µl bacterial culture supernatants into 1 ml BSA buffer (20 mM KH_2PO4, 150 mM NaCl, 1 mM $MgCl_2$, 1 mg/ml BSA) which contains 25 µl rabbit blood cell. All experiments were performed in replicate with two independent repeats. Representative data are shown as mean ± sd. The mean value of hemolytic activity of wild-type strain was set as 100%, the activities of *ΔccpA* and *ccpA::ccpA*^{2CS} mutant were normalized to that of wild-type strain. The statistical difference was determined by two-tailed Student's *t*-test.

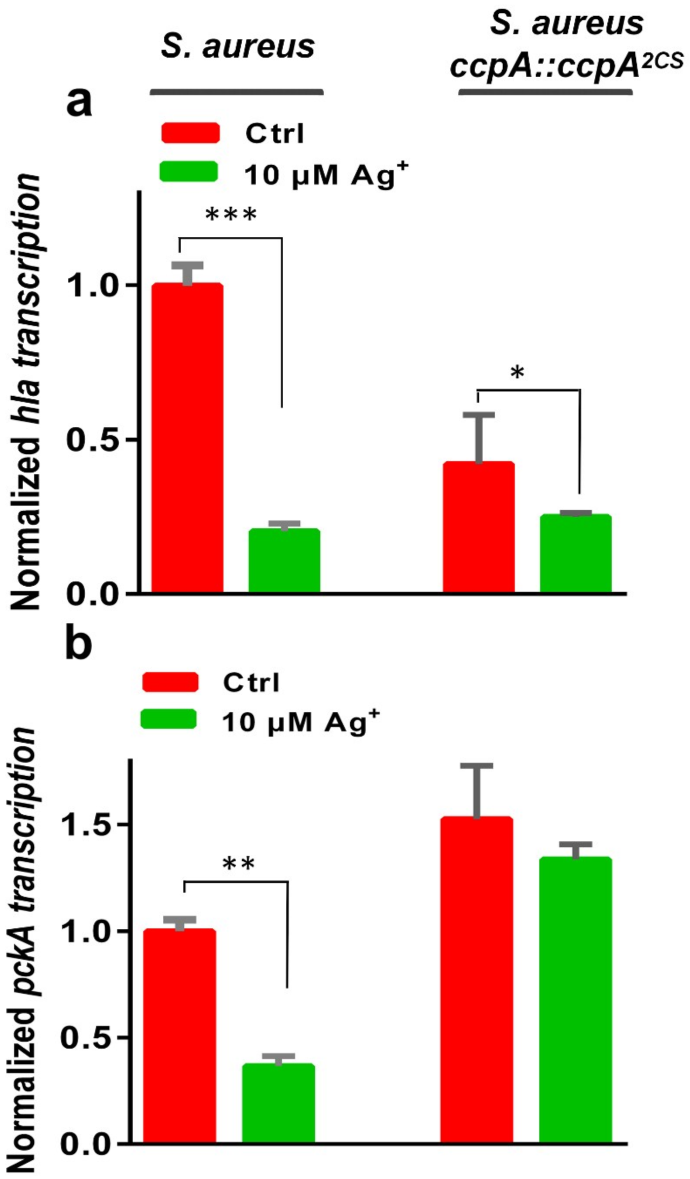

Fig. S12 Quantitative transcript analysis of *hla* (**a**) and *pckA* (**b**) of wild-type *S. aureus* and *S. aureus ccpA::ccpA^{2CS}* mutant with or without Ag^+ treatment. All experiments were performed in triplicate. Results are shown as mean ± sd. The mean value of transcription level in wild type *S. aureus* control groups was set as 1. The transcription levels in other groups were normalized to that of control group. The statistical difference is determined by two-tailed Student's *t*-test.

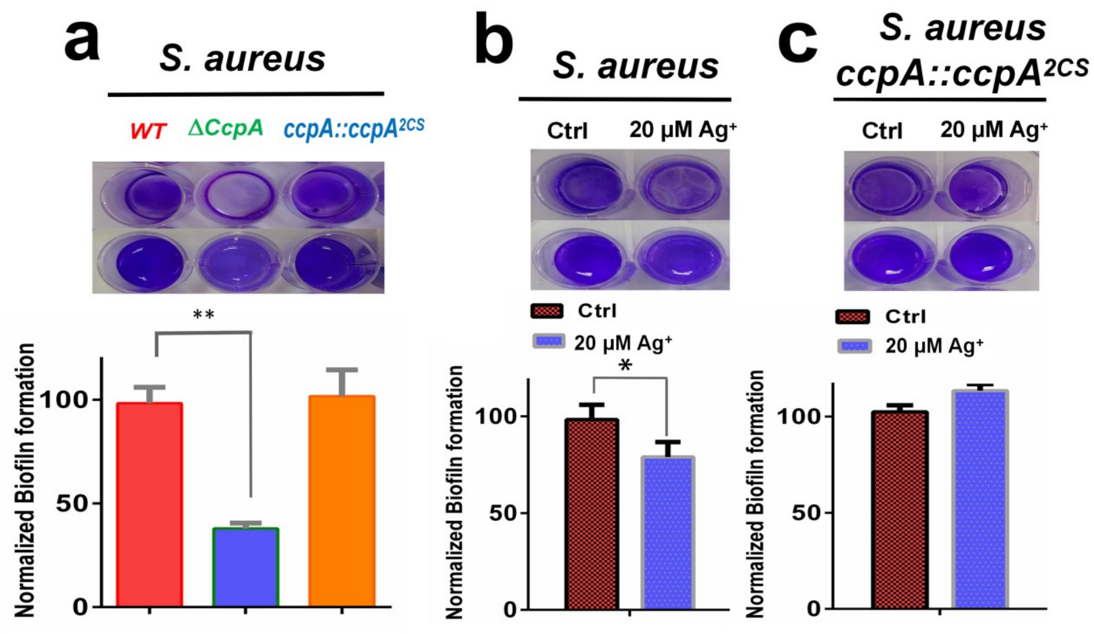

Fig. S13 The biofilm formation of *S. aureus, S. aureus ΔccpA* and *S. aureus ccpA::ccpA^{2CS}* strains **(a)**. The effect of 20 μM Ag^+ on the biofilm formation of wild-type *S. aureus* **(b)** and *S. aureus ccpA::ccpA^{2CS}* **(c)** strains. All experiments were performed in replicate with two independent repeats. Representative data are shown as mean ± sd. The mean value of control group was set as 100%, the absorbance in other groups were normalized to the mean value of control group. The statistical difference is determined by two-tailed Student's *t*-test.

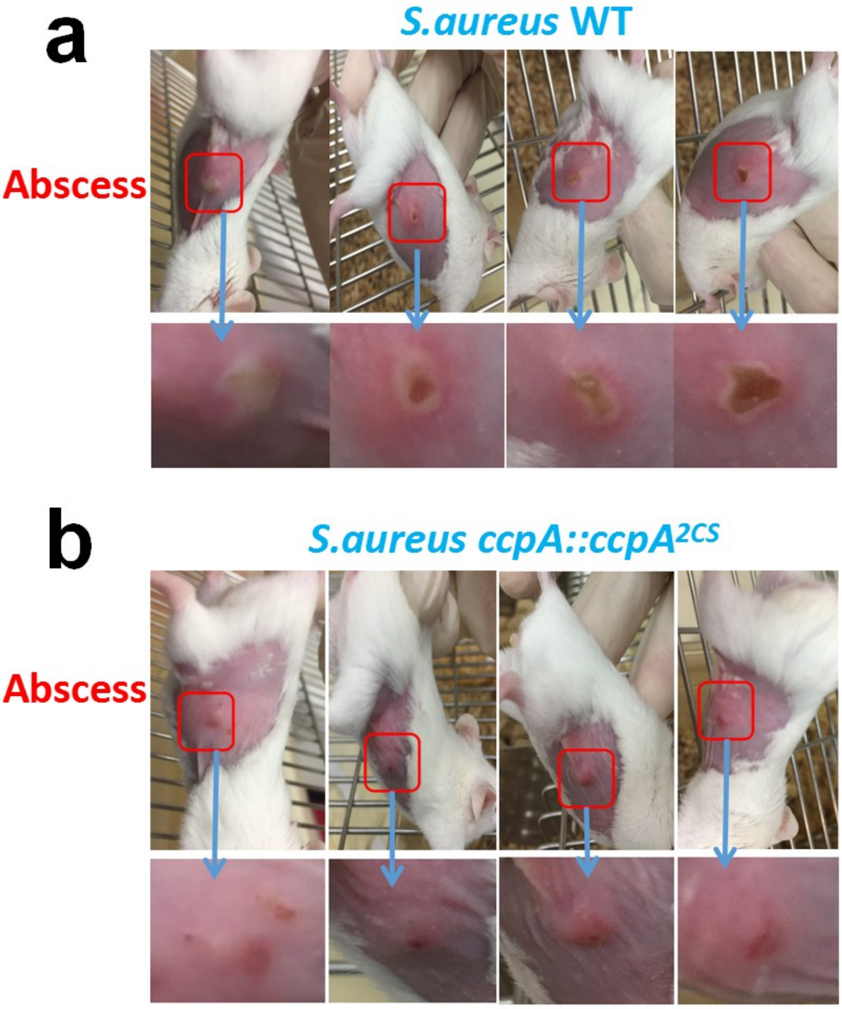

Fig. S14 Murine skin infection model showed the difference on mice abscesses infected with wild- type *S. aureus* (**a**) and *ccpA::ccpA*^{2CS} mutant (**b**).

Table S1 Strains, plasmids and primers

Strain, plasmids or primers	Application	
***E.coli* strains**		
XL1-Blue	Plasmid maintenance	
BL21(DE$_{3)}$	Protein expression	
***Staphylococcus aureus* strains**		
RN4220	Modification plus strain	
Newman	Wild-type strain. Used in CETSA, biofilm and erythrocyte lysis assay	
Newman *ΔccpA*	*ccpA* gene knockout strain. Used in biofilm and erythrocyte lysis assay	
Newman *ccpA::ccpA^{2CS}*	Wild-type *ccpA* gene was replaced by *ccpA*^{2CS}. Used in CETSA, biofilm and erythrocyte lysis assay	
**Plasmids**		
pET47b		
pET47b-*ccpA*	Wild-type CcpA protein expression	
pET47b-*ccpA*^{C216S}	CcpA^{C216S} mutant protein expression	
pET47b-*ccpA*^{C242S}	CcpA^{C242S} mutant protein expression	
pET47b-*ccpA*^{2CS}	CcpA^{2CS} mutant protein expression	
pET32a-*hprK*	His$_6$-HPrK/P protein expression	
pET47b-*hpr*	His$_6$-HPr protein expression	
pKOR1	*S. aureus* mutants construction	
**Primers for CcpA, CcpA mutants, HPr and HPrK**		
	**Forward Primer**	**Reverse Primer**
CcpA	TAGCTCATATGACAGTTACTATATAT<br>GATGTAGCAAGAGAAGCGCG	TATGGATCCTTATTTTGTAGTTCCTC<br>G<br>GTATTCAATTCTGTGAGG
CcpA^{C216S}	CTTCGTCGCTGATACTTAAAATGGCA<br>TCTGGCAAATTGCC	GGCAATTTGCCAGATGCCATTTTAA<br>GTATCAGCGACGAAG
CcpA^{C242S}	CTTTCAGCACCAGAACTATTCAATGTAT<br>CACCTAATTGAAGGCC	GGCCTTCAATTAGGTGATACATTGA<br>ATAGTTCTGGTGCTGAAAG
HPr	TATAGAATTCATGTTAACGACAGAAAA<br>ACTAGTTGAAACAT	TATAAAGCTTCTACTCCTCACTCTTA<br>TGACTGTTC
HPrK	TATAGAATTCTATGGAACAAAATTCAT<br>ATGTAATCATCGAC	TATAAAGCTTTTATTTAGTCAATCCT<br>TCTTTTGATAAGAC
**Primers for EMSA**		
pckA	GCTGGGTTTCATTGGGTCCATGTCCCT	CAGTGTATGTGTCTACTGACATCCTA<br>AATCCC
hla	CATAATTAATACCCTTTTTCTCTATTTCT<br>ATTTATTAATTT	TAGCAACTGATAAATTACTGAGTGA<br>TGATGAGTGAT

proC	ACTCGTACCACCAGTCACTATAA	CGAGTTTCATTAACGTCACATCC
**Primers for qRT-PCR**		
16s RNA	CCATAAAGTTGTTCTCAGTT	CATGTCGATCTACGATTACT
pckA	CGTTGGTGAAAAAGGCGATGT	GCATAGCAGCCACCTTCGAT
hla	AACCCGGTATATGGCAATCAACT	CTGCTGCTTTCATAGAGCCATTT
**Primers for *S. aureus* mutants construction**		
pKOR1-*ΔccpA* up	GGGGACCACTTTGTACAAGAAAGC TGGGTATATGCAAAACTTCGAGTT ATTAAAAGAAG	AAAGGATCCGTTTCTGCTTTAAC ATTTTGGTTCCC
pKOR1-*ΔccpA* down	ATTGGATCCAGGGATGCGCTTATT AACAAAATATATG	GGGGACAAGTTTGTACAAAAAA GCAGGCTGGATTAAATGATGAA GAGAATGGTCAAT
pKOR1-*ccpA::ccpA*^{2CS}	ATTGGTACCAAATATGCAAAACTT CGAGTTATTAAAAGAAG	ATTGGATCCGGATTAAATGAT GAA GAG AAT GGT CAA T

Table S2 Isothermal titration calorimetry data

Protein	Ligand	K_d (μM)	N	ΔH (kcal mol^{-1})	ΔS (cal mol^{-1} k)
**CcpA**	Ag^+	0.74±0.03	1.94±0.02	-7.61±0.31	2.53
**CcpA^{C242S}**	Ag^+	7.81±0.61	1.08±0.03	-9.64±0.37	-8.97
**CcpA^{2CS}**	Ag^+	No binding			

Table S3 Bio-layer interferometry assay data

Sensor	Protein	Binding Model	K_D (M)	K_{on} (1/ms)	K_{off} (1/s)
SA-pckA	**CcpA**	**1:1**	1.61E-08	1.13E+05	1.81E-03
SA-pckA	**CcpA +Ag^+**	**No binding**			
SA-pckA	**CcpA^{2CS}**	**1:1**	1.51E-08	3.71E+05	5.59E-03
SA-pckA	**CcpA^{2CS}+Ag^+**	**1:1**	2.07E-08	3.80E+05	7.86E-03

1. L. Zhang, N. Li, K. Cao, X. Yang, G. Zeng, X. Sun and Q. He, *J. Inorg. Biochem.*, 2016.
2. L. G. J. Nijtmans, N. S. Henderson and I. J. Holt, *Methods*, 2002, **26**, 327-334.
3. T. Bae and O. Schneewind, *Plasmid*, 2006, **55**, 58-63.
4. J. L. Bose, P. D. Fey and K. W. Bayles, *Appl. Environ. Microbiol.*, 2013, **79**, 2218-2224.
5. K. Yasunaka, F. Abe, A. Nagayama, H. Okabe, L. Lozadaperez, E. Lopezvillafranco, E. Muniz, A. Aguilar and R. Reyeschilpa, *J. Ethnopharmacol.*, 2005, **97**, 293-299.
6. J. L. Rodrigueztudela, F. Barchiesi, J. Bille, E. Chryssanthou, M. Cuencaestrella, D. W. Denning, J. P. Donnelly, B. Dupont, W. Fegeler and C. B. Moore, *Clin. Microbiol. Infect.*, 2003, **9**, 1-8.
7. J. Qiu, X. Niu, J. Dong, D. Wang, J. Wang, H. Li, M. Luo, S. Li, H. Feng and X. Deng, *The Journal of Infectious Diseases*, 2012, **206**, 292-301.
8. J. Qiu, X. Zhang, M. Luo, H. Li, J. Dong, J. Wang, B. Leng, X. Wang, H. Feng and W. Ren, *PLOS ONE*, 2011, **6**.
9. D. Cue, J. M. Junecko, M. G. Lei, J. S. Blevins, M. S. Smeltzer and C. Y. Lee, *PLOS ONE*, 2015, **10**.
10. Y. Ma, Y. Xu, B. D. Yestrepsky, R. J. Sorenson, M. Chen, S. D. Larsen and H. Sun, *PLOS ONE*, 2012, **7**.
11. B. Amorena, E. Gracia, M. Monzon, J. Leiva, C. Oteiza, M. Perez, J. L. Alabart and J. Hernandezyago, *J. Antimicrob. Chemother.*, 1999, **44**, 43-55.
